# *RHO*-Associated Retinitis Pigmentosa: Genetics, Phenotype, Natural History, Functional Assays, and Animal Model – In Preparation for Clinical Trials

**DOI:** 10.1167/iovs.66.9.69

**Published:** 2025-07-30

**Authors:** Malena Daich Varela, Juan Carlos Romo-Aguas, Rosellina Guarascio, Kalliopi Ziaka, Monica Aguila, Kwan-Leong Hau, Yumei Li, Rui Chen, Angelos Kalitzeos, Anthony G. Robson, Rebecca A. Baker, Omar A. Mahroo, Andrew R. Webster, Henry Chan, Nathan B. Lubock, Matthew L. Albert, Michael E. Cheetham, Michel Michaelides

**Affiliations:** 1 Moorfields Eye Hospital, London, United Kingdom; 2 UCL Institute of Ophthalmology, University College London, London, United Kingdom; 3 Gavin Herbert Eye Institute - Center for Translational Vision Research, Department of Ophthalmology, University of California Irvine School of Medicine, Irvine, California, United States; 4 Octant, Inc., Emeryville, California, United States

**Keywords:** rhodopsin, inherited retinal dystrophies (IRDs), genetic diseases

## Abstract

**Purpose:**

The purpose of this study was to describe the largest cohort of *RHO*-associated retinitis pigmentosa (RP) to date, analyzing the spectrum of phenotypes, variants, disease natural history, and genotype-phenotype correlations.

**Methods:**

Variants were classified using functional assays, animal models, and published data. Clinical assessments involved visual acuity (LogMAR), dilated fundus examinations, multimodal imaging (spectral-domain optical coherence tomography [SD-OCT] and fundus autofluorescence [FAF]), and international-standard electrophysiology. Cases were described as having generalized RP or sector RP according to fundus examination and imaging data. Longitudinal analysis evaluated progression rates of visual and structural parameters.

**Results:**

Two hundred patients (140 families) with likely disease-causing variants in *RHO* were identified. Positive family history was documented in 78.5% of the cases. Generalized RP was diagnosed in 64%, sector RP in 34.5%, and 1.5% were asymptomatic carriers. Fifty-six variants were identified, 54% were classified as class 2, 14% as class 1, 5% as class 4, and 2% as class 3. Variants in class 1 were associated with earlier symptom onset (mean = 13.5 years), generalized RP, and the worst baseline visual acuity (mean LogMAR = 0.45). Pro347Leu was the most prevalent variant (17%). Longitudinal analysis showed slower progression in sector RP (0.01 LogMAR/year) compared to generalized RP (0.03 LogMAR/year). Imaging revealed distinct phenotypes, including choroideremia-like features in generalized RP and inferior retinal involvement in sector RP that an animal model suggests is light related.

**Conclusions:**

*RHO*-associated RP encompasses a wide phenotypic spectrum with distinct genetic subtypes influencing disease severity and progression. These findings provide critical insights for patient counseling, identifying clinical endpoints, participant stratification, and guiding therapeutic development.

Retinitis pigmentosa (RP) encompasses a class of hereditary retinal dystrophies characterized by progressive rod-cone dystrophy, night blindness, visual field narrowing, and often eventual decreased visual acuity.^1^^,^^2^ With a prevalence of approximately 1 in 3000/4000 individuals worldwide, RP poses a significant burden on affected individuals, their families, and society, especially given the limited therapeutic options currently available.^3^^,^^4^

Rhodopsin (encoded by the *RHO* gene, MIM *180380) is the primary visual pigment in rods and a G protein-coupled receptor that represents the first step in the visual transduction cascade.^5^^–^^9^ The seminal discovery of its association with RP marked a watershed moment in the field of ophthalmic genetics.^10^ *RHO* was the first gene implicated in RP and this finding provided a foundation for understanding the molecular mechanisms of RP and initiated the identification of the complex genetic architecture of inherited retinal dystrophies (IRDs).

Variants in *RHO* are among the most common causes of RP, and the most frequent cause of autosomal dominant (AD) and sector RP.^11^^,^^12^ They have also been rarely associated with AD congenital stationary night blindness^10^ and autosomal recessive RP.^13^

In this paper, we describe the largest cohort to date of *RHO*-associated RP, the spectrum of phenotypes and likely disease-causing variants, the natural history, and the molecular mechanisms underlying this retinal degeneration. Furthermore, we discuss the potential therapeutic strategies targeting *RHO* and its associated pathways, highlighting the challenges and opportunities in the pursuit of new treatments.

## Methods

### Study Design and Participants

This report is a retrospective consecutive case series of patients who attended Moorfields Eye Hospital (MEH, London, UK), were diagnosed with or had a positive family history of IRD, and were found to have a rare or likely disease-causing variant in *RHO* through targeted Sanger sequencing, panel-based targeted next generation sequencing, exome or whole genome sequencing (WGS). Patients were identified through the inherited eye disease database at MEH. DNA was extracted from whole blood. When appropriate and available, samples were taken from parents or siblings to confirm familial segregation. The pathogenicity of each variant was classified according to the guidelines of the American College of Medical Genetics and Genomics (ACMG) and *RHO* transcript ID used was NM_000539.3.^14^^–^^16^ Informed consent was obtained from all patients. Ethical approval was provided by the local ethics committee, and the study honored the tenets of the Declaration of Helsinki.

### Clinical Assessments

Relevant patient data were retrieved from the electronic healthcare records and imaging software systems. Snellen best corrected visual acuity (BCVA) was recorded and converted to LogMAR for statistical purposes. Count fingers vision was taken to correspond to LogMAR 1.98, hand motion to LogMAR 2.28, light perception to LogMAR 2.7, and no light perception to LogMAR 3.0. Asymmetric BCVA was defined as a difference ≥ 0.3 LogMAR (equivalent to 15 Early Treatment Diabetic Retinopathy Study [ETDRS] letters) between eyes. Patients were categorized using the World Health Organization (WHO) visual impairment criteria, which defines no or mild visual impairment as BCVA ≤ 0.48 (6/18 or 20/60), moderate impairment as BCVA > 0.48 and ≤ 1.0 (6/60 or 20/200), severe as BCVA > 1.0 and ≤ 1.3 (3/60 or 20/400), and blindness as BCVA > 1.3. Only BCVA was taken into consideration to classify patient visual impairment. BCVA-based legal blindness was defined as BCVA > 1.0 LogMAR in the best seeing eye. Mild refractive error was defined as < 3 diopters (D), moderate ≥ 3 D and < 6 D, and high ≥ 6 D (hyperopia + or myopia –).

Clinical assessments consisted of dilated fundus examination, spectral-domain optical coherence tomography (SD-OCT; Heidelberg Spectralis, Heidelberg Engineering, Inc., Heidelberg, Germany), fundus autofluorescence (FAF; Heidelberg Spectralis, Heidelberg Engineering, Inc., Heidelberg, Germany, and Optos PLC, Dunfermline, UK), and ultrawide field pseudocolor fundus photography (Optos PLC). Patients were described as having generalized or sector RP on the basis of fundus appearance, imaging data, or medical records. OCT thickness in the general population was extracted from Invernizzi et al.^17^ The ellipsoid zone (EZ) width was measured at the foveal scan and was considered as the subfoveal continuous EZ line. The outer nuclear layer thickness (ONLT) was measured in the center of the foveal scan in all eyes. Photoreceptor outer segment length (PROS) was obtained by measuring the distance between the inner border of the EZ and the inner border of retinal pigment epithelium (RPE) in the foveal scan, below the foveal pit, trying to capture the hyper-reflective “peak to peak.”^18^ PROS were measured by two independent observers (authors M.D.V. and J.C.R.A.). The values presented are an average of both measurements and the difference between both observers was between 0 and 7 µm (median 1 µm = 7%). Only blue autofluorescence (BAF; Heidelberg) was used for macular hyperautofluorescent ring quantitative assessment. Given the shorter axial length in younger patients and the impact this has on transverse measurements, EZ width (EZW) and BAF ring measurements were also calculated excluding patients under 20 years of age. Thickness of ONL and PROS length were not adjusted as these are not affected by axial length.^19^

Pattern and full-field electroretinogram (PERG and ERG) testing was performed in a subset of patients, incorporating the standards of the International Society for Clinical Electrophysiology of Vision (ISCEV).^20^^–^^22^ Gold foil corneal electrodes were used in all but one case, tested with silver thread electrodes. Light stimuli were generated using Xenon or LED-based Ganzfeld stimulators. The main components of the ISCEV dark-adapted (DA) and light-adapted (LA) ERGs were quantified and compared with age-matched control data from healthy subjects (age range = 10–79 years), specific for the electrode type and stimulator system used. The ERG amplitudes were plotted as a percentage of the age-matched lower limit of the reference range, and peak times plotted as a difference from the age-matched upper limit of the reference range.^23^^,^^24^

### Variant Classification

**Figure 1.**
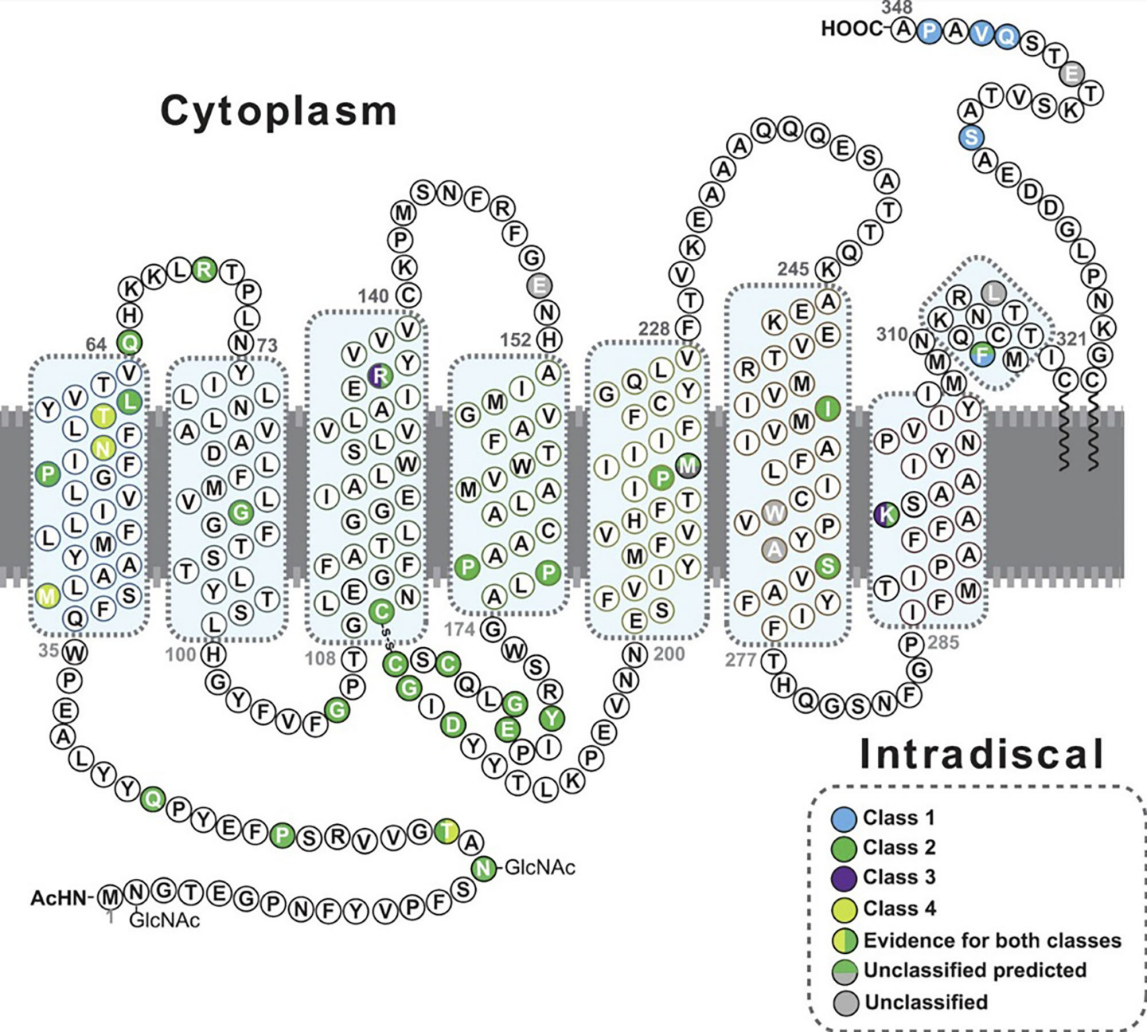
The position of amino acid residues affected by *RHO* variants found in this cohort are represented in the protein's secondary structure to show the distribution of different variant classes. Class 1, post-Golgi trafficking and outer segment targeting (*blue*). Class 2 misfolding, ER retention, and instability (*green*). Class 3, hyperphosphorylation, disrupted vesicular trafficking, and endocytosis (*violet*). Class 4, altered post-translational modifications and reduced stability (*yellow*). Where there is evidence for more than one type of class it is shown with a vertical color split. Uncategorized variants, or those with no observed biochemical or cellular defects, are shown in *grey*. Those with predicted effects are shown with a horizontal color split. The eight alpha helix motifs of rhodopsin are highlighted by *blue boxes*.

Where possible, each variant was assigned to one of the classes suggested by Athanasiou et al.,^6^ based on previous studies of the consequences of the variant on heterologous expression and/or animal models, as well as the HEK293T cell deep mutational scanning (DMS)^25^ and targeted mutagenesis surface trafficking data (Fig. 1, Supplementary Fig. S1A, Supplementary Table S1).

Class 1 variants affect the targeting of rhodopsin to the outer segment (OS); class 2 misfold and are retained in the endoplasmic reticulum (ER); class 3 are hyperphosphorylated and are proposed to affect endocytosis through aberrant arrestin binding; and class 4 are able to exit the ER and traffic to the OS but have reduced stability and disease that is exacerbated by light. In general, if there were reports of a variant being retained in the ER and not trafficking to the plasma membrane on heterologous expression, or a score of < 50% wild-type (WT) rhodopsin trafficking in the DMS or luciferase assay, they were assigned to class 2. By contrast, > 50% or near-WT trafficking levels in heterologous systems was assigned to class 1 if the variant affected the known C-terminal OS targeting motif (VxPx) through substitution, frameshift, or premature termination from helix 8 to the C-terminus. Splice site variants that affect the terminal exon, which encodes helix 8 and the C-terminus, were classified as class 1/2, as there are no experimental data on these variants and it is unlikely they could traffic correctly to the OS, but they could also misfold. Other WT-like trafficking variants with reduced stability or increased light sensitivity were assigned to class 4, with the exception of p.Thr17Met, which was classified at class 2/4 because of evidence of increased light sensitivity in animal models, as well as misfolding in the DMS assay.^25^ Variants at residue Arg 135 and Lys 296 were assigned to either class 3 or class 2/3 if there was also evidence of misfolding. Variants with WT-like trafficking in cell culture systems and no other strong supporting evidence for potential mechanism were unclassified. Where there were conflicting published data on the variant effect this is noted in Supplementary Table S1 and shown in Figure 1, for example, p.R135W, which has been reported as both class 2 and class 3. Where there were conflicting data between heterologous expression and animal model phenotype, more weight was given to the animal model data. Overall, 91% of the variants represented in the cohort could be assigned to a particular variant class.

The RHO trafficking DMS provided functional data for several previously unstudied missense variants to support their categorization as likely class 2. Novel duplication and deletion variants were created using site-directed mutagenesis of the DMS assay construct and studied using the luciferase reporter to determine their effect on trafficking and potential as class 2 variants (see Supplementary Fig. S1).

### Statistical Analysis

Statistical analyses were performed using GraphPad Prism 8.0.2 (GraphPad Software, San Diego, CA, USA). The threshold of significance was set at *P* < 0.05. Analysis included linear regressions, correlations, *t*-tests, and Kaplan-Meier survival to assess various characteristics of the cohort.

## Results

### Cohort Demographics, Phenotype, and Disease Onset

Two hundred patients from 140 families were found to have a likely disease-causing variant in *RHO* (Table 1, Supplementary Table S2)*.* Ninety-one (45.5%) were male patients and 109 (54.5%) were female patients. One hundred seventeen of the patients were White (58.5%), 11 were Asian (5.5%), 7 were Black (3.5%), and 2 were of mixed race (1%), and there were no available data of the remaining 63 patients (31.5%). One hundred fifty-seven (78.5%) patients had a positive family history of an IRD (for 76 it was their father, for 66 it was their mother, and for 15 it was other relatives, for example, maternal uncle, paternal aunt, and maternal great aunt). Ninety-six patients (48%) had familial segregation analysis, 91 (45.5%) carried the familial variant, and 5 patients (2.5%) had confirmed de novo variants.

**Table 1.** Clinical Characteristics of Patients With *RHO*-Associated Retinal Dystrophy

Patients (*n* = 200^*^)	Generalized RP (*n* = 128)	Sector RP (*n* = 69)
Families, *n*	87	51
Gender, *n* (%)		
F	68 (53)	38 (55)
M	60 (47)	31 (45)
Population, *n*		
Asian	9	2
White European	73	44
African descent	6	1
Mixed	2	0
Age of onset, mean ± SD, y	16.1 ± 12.8	28.8 ± 14.8
First symptom	Nyctalopia (86%)	Nyctalopia (55%)
Age at first examination, mean ± SD, y	34.7 ± 17.3	39.5 ± 14.8
Age at last examination, mean ± SD, y	45.2 ± 18.9	47.6 ± 17
Baseline BCVA, mean ± SD, LogMAR	0.35 ± 0.47 OD; 0.4 ± 0.5 OS	0.13 ± 0.33 OD; 0.14 ± 0.4 OS
Baseline area of macular hyperautofluorescent ring, mean ± SD, mm^2^	7.6 ± 6.2 OD; 8.1 ± 7.2 OS	6.6 ± 4.4 OD; 6.9 ± 4.3 OS
Baseline central macular thickness, mean ± SD, µm	250 ± 50	278.5 ± 32
Baseline outer nuclear layer thickness, mean ± SD, µm	87.5 ± 39.5	104 ± 23
Ellipsoid zone width, mean ± SD, mm	2.0 ± 1.3	2.6 ± 1.2
Baseline photoreceptor outer segments, mean ± SD, µm	12.5 ± 15.5	17.5 ± 19
Patients with cystoid macular edema, *n* (%)	43 (33.5)	22 (33)

* Including asymptomatic carriers.

One hundred twenty-eight of the patients (64%) had generalized RP, 69 (34.5%) had sector RP, and 3 (1.5%) were unaffected carriers. Among the individuals with generalized RP (*n* = 128), 8 were asymptomatic (ages 7–40 years old) and the remaining patients had a mean age of onset of 16.3 ± 14.4 years old (birth to 65 years old), with 110 (86%) reporting nyctalopia as the initial complain, and fewer mentioning visual field defects (2%), blurred central vision (2%), or slow adjustment to dark environments (1%).

Of those with sector RP (*n* = 69), 8 were asymptomatic (ages 9–79 years old), and the rest developed symptoms at 30.4 ± 15.7 years old (range = 5–68 years old). The most common symptoms were nyctalopia in 38 patients (55%) and visual field defect in 14 patients (20%); the less frequent symptoms were decreased central vision (3%), photosensitivity (1%), and prolonged adaptation to dim lighting (1%). The three patients who were unaffected carriers did not display retinal symptoms or features by fundus examination and imaging at ages 17, 34, and 46 years, respectively; the latter 2 also had normal ERG and PERGs at ages 34 and 45 years.

There was a statistically significant difference between the age of symptom onset of generalized (16.3 ± 14.4 years old) and sector RP (30.4 ± 15.7 years old, *t*-test *P* < 0.0001). There were no significant differences between male and female patients in age of onset, age at baseline and final visit, initial or final BCVA (*P* = 0.1–0.9), or in the proportion of generalized versus sector RP phenotype. Similarly, no significant differences were observed in these parameters depending on whether the pathogenic allele was maternally or paternally inherited (*P* = 0.4–0.8).

### Molecular Diagnosis and Genotype-Phenotype Correlations

#### Sequence Variants

Fifty-six different variants were present in the cohort; 44 missense, 3 in-frame deletions, 2 nonsense, 2 splice-site, 2 deletion-insertions, 1 in-frame duplication, 1 frameshift duplication, and 1 protein extension (position of the affected residues in the protein secondary structure depicted in Fig. 1, Supplementary Table S1, Table 2). Four variants were not previously reported and all classified as likely pathogenic (7%, c.999dupC, c.937-24_943delins150, c.793T>G [p.Trp265Gly], and c.805_807dupGCC). Only one variant (c.953_955delTCA) was classified as a variant of uncertain significance (VUS). This variant, reported in ClinVar (984770) as both a VUS and likely pathogenic variant, was present in patient ID 18 with a dominant family history of RP, and was included in the cohort and considered a likely *RHO*-related case. One patient (ID 104) had a biallelic homozygous variant, c.448G>A, p.Glu150Lys, a change previously associated with recessive disease.^13^ Another patient (ID 84) was heterozygous for this c.448G>A variant, without any other IRD candidate variant found by WGS. Her phenotype corresponded to a late onset, mild sector RP, and the variant was absent in the unaffected mother; hence, she was included in the cohort as a likely AD *RHO*-related case.

**Table 2.** Genotype-Phenotype Correlation Per Variant and Class

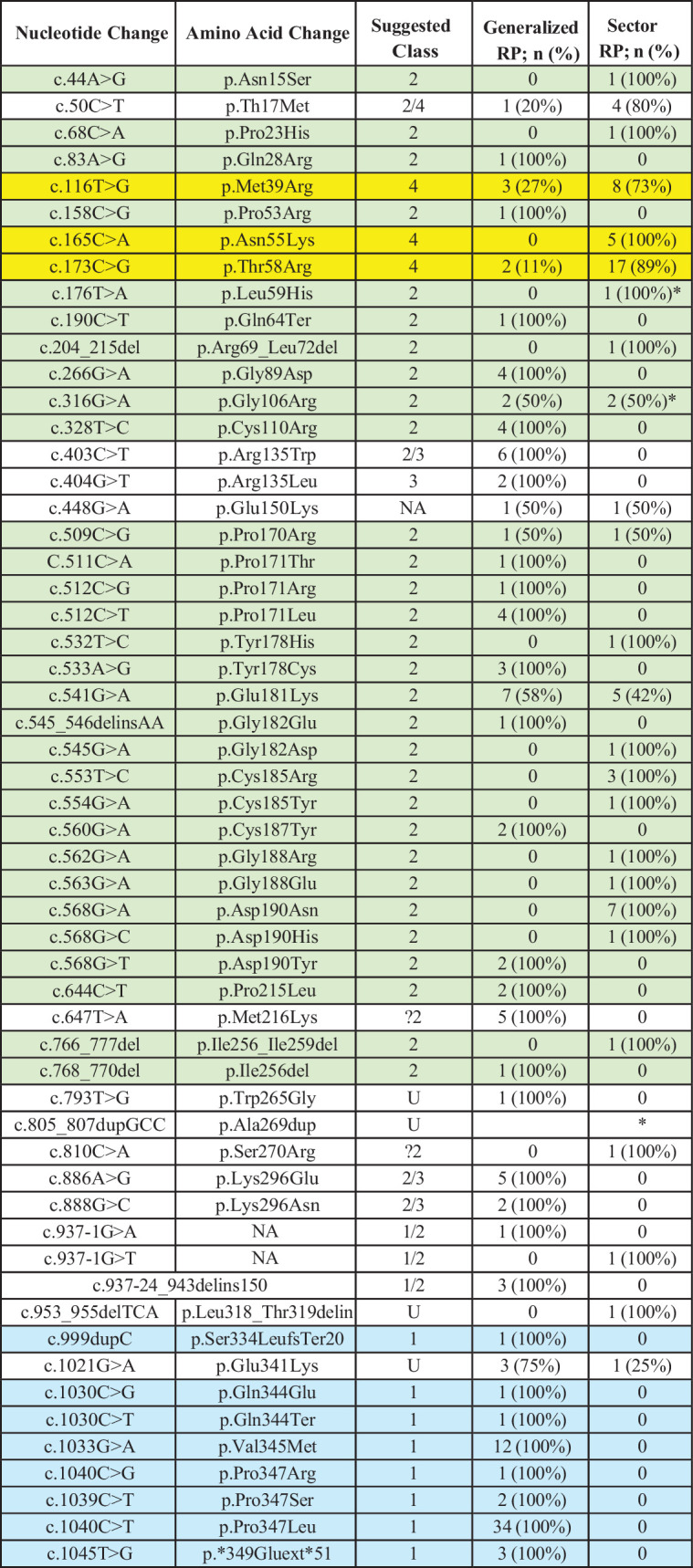

* Represents +1 healthy carrier.

Blue = class 1; green = class 2; yellow = class 4.

The most common variant was c.1040C>T, p.Pro347Leu, found in 24 families (34 patients, 17%; see Table 2), mostly in White ethnicity, and in a minority of Asian and Black patients, all of whom had generalized RP. This was followed by c.541G>A, p.Glu181Lys and c.173C>G, p.Thr58Arg in 11 families (8% each) of White ethnicity. Patients with p.Glu181Lys had both generalized and sector phenotype (6 and 5 patients, respectively), whereas those with p.Thr58Arg had a majority of sector RP (17/19 patients). A knock-in mouse model of the p.Thr58Arg variant showed a light induced retinal degeneration (Supplementary material and Supplementary Fig. S2), consistent with a role of light exposure in sector RP.

#### Mutation Class and Phenotype Correlations

Eight variants (14%) were classified as class 1, there were 30 (54%) as class 2, there was 1 (2%) as class 3, there were 3 (5%) as class 4, there were 2 (4%) as possibly class 2, and 7 (12.5%) as mixed with class 2 (three in class 1/2, 3 in class 2/3, and 1 in class 2/4, with evidence supporting different potential effects depending on the model system; see Fig. 1, Supplementary Table S1). The remaining five variants (9%) remained unclassified due to lack of experimental evidence. The variant c.190C>T, p.Gln64Ter in patient ID 59 is the only one which could possibly act as null, but has been reported to escape nonsense mediated decay and the truncated protein does not fold correctly^26^^,^^27^; therefore, it was designated as class 2. For statistical and group genotype-phenotype correlation analyses, only patients with variants in one defined class were considered.

Fifty-five patients (27.5%) had variants in class 1 and their phenotype was generalized RP in all cases. Seventy patients (35%) had class 2 variants, being the most common class in our cohort. Of these, 38 patients (54%) had generalized RP, 30 (43%) had sector, and 2 (3%) were unaffected carriers. Class 3 was only composed of 2 individuals with a generalized RP phenotype. Class 4 was composed of 35 individuals (17.5%), of whom 5 had generalized RP (14%) and 30 (86%) had sector RP. Thirteen patients (6.5%) had variants that fit as a combined class 2 and 3 (2/3) and all had generalized RP. Five patients (2.5%) had variants of combining class 1 and 2 (1/2); and 4 of these had generalized RP and 1 had sector RP. Five combined class 2 and 4 (2/4; 2.5%), 4 with sector RP and 1 with generalized. Last, 6 patients had variants classified as potential class 2 (3%, ?2 in Supplementary Table S2), 5 patients with generalized RP, and 1 patient with sector RP. Nine patients (4.5%) had variants that did not fit into a known variant class. A significant association was observed between RP phenotype and variant class (Chi² = 68.6, *P* < 0.0001), with class 1 highly enriched in generalized RP (standardized residual = +3.6), class 4 in sector RP (+4.6), and class 2 with a more balanced distribution without significant deviations from expected frequencies. Patients with variants in class 1 were approximately 1.8 times more likely to have generalized RP compared to class 2, and 7 times more likely compared to class 4. Additionally, patients in class 2 were approximately 3.9 times more likely to have generalized RP than those in class 4.

Mean age of symptom onset was earliest for class 1 (13.5 ± 7.7, median = 13), followed by class 2 (24 ± 17, median = 16), and last class 4 (29.2 ± 14.9, median = 26); whereas the 2 patients in class 3 were asymptomatic (both seen during childhood). The patients in class 1/2 had a disease onset between the second and third decades of life (17.3 ± 6.7 years old, median = 14), patients in class 2/4 were between the second and fourth decades of life (23.2 ± 7 years old, median = 24), and those patients in class 2/3 had the earliest onset, between the first and second decades (9 ± 3.3, median = 10), and last those in ?2 had a later onset, between the third and sixth decades of life (45 ± 13.9, median = 47).

### Visual Acuity and Anterior Segment

#### Generalized RP

The mean age at the first visit was 34.7 ± 17.3 years old (range = 3–80 years old). Baseline BCVA was 0.35 ± 0.47 OD and 0.4 ± 0.5 OS (median = 0.2 OU), with 101 patients (79%) having no or mild visual impairment based on BCVA, 18 (14%) had moderate impairment, 2 (2%) had severe impairment, and 5 (4%) were blind. Baseline BCVA was not available in two patients. Asymmetric BCVA was observed in 17 patients (13%). Seventy-seven patients (60%) had cataracts or were pseudophakic at a mean age of 44.9 ± 12.3 years old.

#### Sector RP and Asymptomatic Carriers

Mean age at the first visit was 39.5 ± 14.8 years old (range = 9–78 years old). Baseline BCVA was 0.13 ± 0.33 OD and 0.14 ± 0.4 OS (median = 0.0 OU), with 68 patients (94%) having no or mild visual impairment based on BCVA, 3 (4%) having moderate impairment, and 1 (1%) being blind. Asymmetric BCVA was seen in nine patients (12.5%). Fourteen patients (20%) had cataracts or were pseudophakic at a mean age of 52.8 ± 15.2 years old.

The 3 asymptomatic carriers were evaluated due to family history and had a baseline visit at ages 16, 29, and 44 years. The baseline BCVA was normal for 2 patients and 0.2 OD to 0.5 OS in the third, who experienced left acute anterior uveitis that later improved.

Analyzed cross-sectionally at the initial visit, there was a significant association between age and BCVA OD and OS in the generalized RP group (*P* = 0.0085 and 0.0016, *R*^2^ = 0.06). In patients with sector RP, there was a significant association only in BCVA OD (*P* = 0.01, *R*^2^ = 0.09), but not in OS (*P* = 0.14). There were statistically significant differences between baseline BCVA in generalized versus sector RP (*P* = 0.01 and 0.007 for OD and OS, respectively).

#### Genotype-Phenotype Correlations

At the baseline visit, class 1 was significantly younger than class 2 (32.6 ± 15.1 vs. 40.6 ± 15.1 years old, *P* = 0.004), whereas no significant differences were found between 1 and 4 (38.1 ± 16.6 years old, *P* = 0.1), or 2 and 4 (*P* = 0.46; Table 3).

**Table 3.** Genotype-Phenotype Correlations in Different Classes of *RHO* Variants

	Class 1 (*n* = 55, 27.5%)	Class 2 (*n* = 70, 35%)	Class 4 (*n* = 35, 17.5%)
Phenotype			
Generalized RP	55 (100%)	38 (54%)	5 (14%)
Sector RP	0	30^*^ (43%)	30 (86%)
Age of onset, y	13.5 ± 7.7	24 ± 17	29.2 ± 14.9
Age at baseline, y	32.6 ± 15.1	40.6 ± 15.1	38.1 ± 16.6
Childhood, 3–12 y old	26	20	4
Adolescence, 13–19 y old	19	14	3
Adulthood, over 19 ye old	8	28	22
Baseline central macular thickness, mean ± SD, µm	241.8 ± 52.4 OD; 251.9 ± 50.9 OS	263.2 ± 45.7 OD; 267.9 ± 49.5 OS	272.4 ± 36.1 OD; 269.6 ± 32.9 OS
Baseline outer nuclear layer thickness, mean ± SD, µm	86.3 ± 36.9 OD; 88.4 ± 43.7 OS	92.7 ± 36.2 OD; 96.2 ± 38.9 OS	94.7 ± 28.8 OD; 94.9 ± 29.2 OS
Ellipsoid zone (EZ) width, mean ± SD, µm	1559.7 ± 1073.1 OD; 1521.2 ± 1089.8 OS	2439.4 ± 1447.2 OD; 2521.8 ± 1380.3 OS	2261.8 ± 1300.5 OD; 2333.7 ± 1195.8 OS
Baseline photoreceptor outer segment length, mean ± SD, µm	11.5 ± 7.9 OD; 10.7 ± 8.8 OS	15.8 ± 8.5 OD; 17.1 ± 7.1 OS	14.6 ± 7.1 OD; 14.9 ± 6.8 OS
Follow up time, y	12.4 ± 10	10.4 ± 9.2	9.7 ± 7.8
Rate of progression BCVA, LogMAR/y	0.025 OD; 0.035 OS	0.02 OD and OS	0.03 OD and OS
Rate of ring constriction, mm^2^/y	0.2 ± 0.2	0.4 ± 0.5	0.3 ± 0.2
Rate of EZ constriction, µm/y	−64 OD; −23 OS	−96 OD; −35 OS	−110 OD; −45.8 OS

* Plus two healthy carriers.

The mean age at baseline visit was 38 years old for all classes (excluding class 3 due to only 2 young members). The mean baseline BCVA was 0.45 ± 0.6 OD and OS for class 1, with 3 individuals being blind and 1 severely sight impaired. In class 2, mean BCVA was 0.2 ± 0.4 OD and 0.3 ± 0.6 OS; 2 patients were blind. In class 4, mean baseline BCVA was 0.2 ± 0.4 OD and OS, and no individual was blind or severely sight impaired.

In class 1, BCVA OD was significantly worse than in class 2 (*P* = 0.039) and both OD and OS were worse compared to class 4 (OD *P* = 0.03 and OS *P* = 0.016). No significant differences were found in BCVA class 1 OS and class 2 OS (*P* = 0.13), or between classes 2 and 4 (*P* = 0.26 and 0.58, respectively).

In class 1, there were 39 patients (71%) who had or developed cataracts during follow up; in class 2, there were 29 patients (41%); and in class 4, there were 8 (23%).

### Visual Electrophysiology

International-standard visual electrophysiology was available in 63 patients tested with corneal recording electrodes. There was a high degree of inter-ocular symmetry in the amplitudes of the ISCEV Standard DA 0.01, DA 10 ERG a- and b-waves, LA 30 hertz (Hz) ERG and LA 3 (single flash) ERG a- and b-waves (slope = 1.0, *r*^2^ = 0.94), pattern ERG P50 components (slope = 0.94, *r*^2^ = 0.88), and in peak times of the DA10 ERG a- and b- waves, LA 30 Hz ERGs and LA 3 ERG a- and b-waves (slope = 0.95, *r*^2^ = 0.87).

Supplementary Figure S3 summarizes the ISCEV Standard ERG and PERG P50 findings and patient ages at the time of testing. All but four patients had full-field ERG abnormalities in keeping with a photoreceptor dystrophy, with relatively greater reduction in the DA 10 ERG a-wave compared with LA ERGs in most cases (see Supplementary Fig. S3), consistent with a rod-cone dystrophy. Six patients with moderate retinal dysfunction showed relatively greater LA ERG b-wave than DA 10 ERG a-wave reduction, suggesting slightly greater cone than rod system involvement (see Supplementary Fig. S3; numbers 40, 46, 47, 49, 50, and 54). Two of the four patients with normal ERGs were asymptomatic carriers, one had clinical evidence of restricted pigment, and one had diffuse fundus changes. Thirteen of 59 patients with DA 10 ERG a-wave reduction had a reduced b:a ratio, including 11 with undetectable DA 0.01 ERGs. Full-field ERG reductions and delays tended to be worst in those with fundus changes in keeping with generalized RP compared with those classified as having sector RP, with findings in these two groups summarized below.

#### Generalized RP

Twenty-nine patients with a fundus phenotype suggestive of generalized disease had ERG testing at a mean age of 30 years (median = 26 years and range = 7 to 75 years); one had undetectable ERGs at age 42 years (case 3; see numbered case 1 in Supplementary Fig. S3). Most others had ERG evidence of rod-cone dystrophy (see Supplementary Fig. S3). In the 24 cases with detectable LA 30 Hz ERGs, peak times were delayed (*N* = 20, median = 8 ms, range = 2–15 ms), in keeping with generalized retinal dysfunction, were borderline (*N* = 2 = ± 0.5 ms of the upper limit of normal) or just within the reference range (*N* = 2). In the 23 cases where pattern ERG data were available, P50 components were undetectable (*N* = 3) or subnormal (*N* = 15), in keeping with macular involvement; and normal in 5 patients (see Supplementary Fig. S3). This group included all six cases that showed slightly greater cone than rod system involvement (see Supplementary Fig. S3).

#### Sector RP

Thirty-two patients with clinical evidence of sector RP had ERG testing at a mean age of 43 years (median = 42 years and range = 18 to 79 years). The ERGs indicated a rod-cone dystrophy in most, generally milder than in those with widespread fundus changes (see Supplementary Fig. S3). LA 30 Hz ERG peak times were delayed in 12 patients (by 1 to 8 ms, median = 2 ms), were borderline (*N* = 6), or were normal (*N* = 15), the latter consistent with restricted or a localized loss of photoreceptor function rather than generalized retinal dysfunction. In the 30 cases where pattern ERG data were available, P50 components were undetectable (*N* = 2), subnormal (*N* = 19), or normal (*N* = 9).

#### Genotype-Phenotype Correlation

The electrophysiological findings associated with different types of variants are illustrated in Supplementary Figure S4, including those classified as type 1 (*N* = 9), type 2 (*N* = 29), type 3 (*N* = 1), type 1/2 (*N* = 1), type 2/4 (*N* = 1), and type 2/3 (*N* = 2), and type 4 (*N* = 10). Class 1 variants were associated with consistently severe ERG reductions, with all 9 cases showing a greater than 50% reduction in both DA 10 ERG a-waves and LA 30 Hz ERGs (see Supplementary Fig. S4). Seven of 7 with detectable LA 30 Hz ERGs showed peak time delays, severe (> 9 ms) in 5 cases. Pattern ERG P50 was undetectable in 1 of 6 cases tested, and was mildly reduced in the 5 others, consistent with relative sparing of macular function. All nine patients had a “generalized” fundus phenotype.

Class 2 variants were associated with wide ERG variability (*N* = 29) with DA 10 ERGs ranging from undetectable to normal, and with LA 30 Hz ERG delays in 13 of 27 cases with a detectable response (range = 1–16 ms). Twelve of the 29 patients with the greatest DA 10 ERG a-wave reductions had a “generalized” fundus phenotype; all others had fundus appearance consistent with sector RP. Pattern ERG P50 also showed wide variability in both fundus phenotype groups (Supplementary Fig. S5).

Class 4 variants were consistently associated with relatively mild DA 10 ERG a-wave reductions (amplitudes reduced to 31%–88% of the lower limit of normal) with consistently milder LA 30 Hz ERG reductions. There were relatively mild LA 30 Hz delays in 4 of 11 cases (median delay = 2.5 ms, range = 2–7 ms) and borderline peak times in 4 cases. The most severe ERG phenotype in this group was seen in the only case associated with generalized RP on fundus examination, with the remaining nine having sector RP. Pattern ERG P50 components were mostly normal (*n* = 4) or mildly reduced (*n* = 4), with only 1 of 10 cases having an undetectable response.

### Fundus Evaluation and Retinal Imaging (Photography and Fundus Autofluorescence)

#### Generalized RP

**Figure 2.**
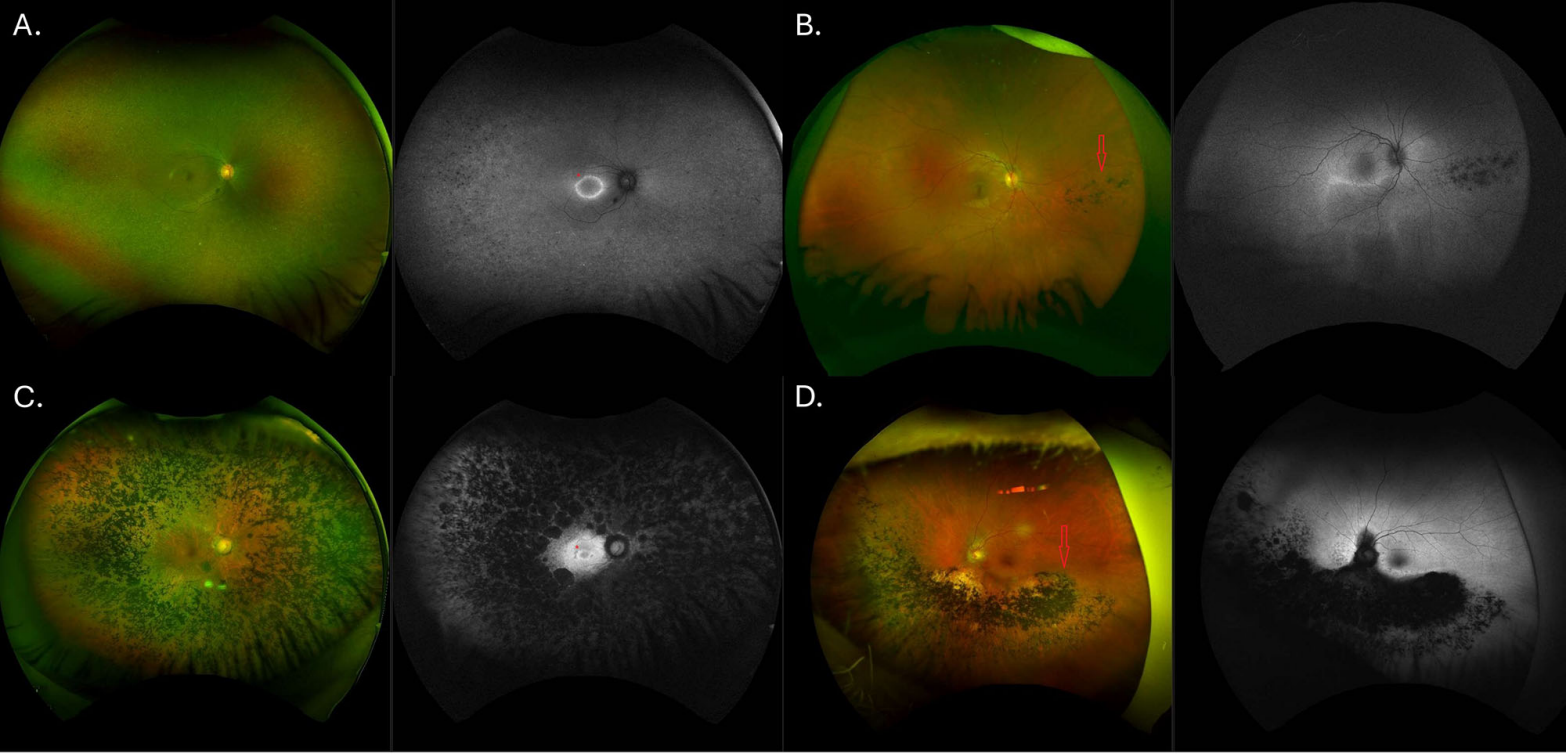
Fundus imaging of patients with *RHO*-associated retinitis pigmentosa (RP). (**A**) Patient ID 5 at 21 years of age with generalized RP. The pseudo-color imaging shows the retinal periphery with a granular pattern, vessel thinning, and minimal pigment deposits, and the autofluorescence (AF) shows peripheral hypoautofluorescent (hypoAF) spots along with a hyperautofluorescent macular ring (marked with *). (**B**) Patient ID 129 at 15 years old with sector RP, with minimal bone spicule-like (BSL) pigment in the nasal retina (marked with a *red arrow*), and a macular hyperautofluorescent inferior rim in the AF image. (**C**) Patient ID 47 at 57 years of age with generalized RP presents with dense peripheral BSL pigment deposits and both diffuse and patchy hypoAF, a relatively preserved macula, and a small hyperautofluorescent macular ring (marked with *). (**D**) Patient ID 22 at 89 years old with sector RP, has advanced atrophy with dense BSL pigments in the nasal and inferior retina, over the inferior vascular arcade, and including the peripapillary region (marked with a *red arrow*), with preserved macula and superior retina. These cases illustrate both phenotypes of *RHO*-associated RP at early and late stages of the condition.

One hundred nine patients (85%) had baseline retinal images at a mean age of 39.7 ± 18.4 years old. Ninety-six patients (75%) had widefield color images with common signs including bone spicule-like (BSL) pigment, mottled/granular appearance, atrophy, and vessel attenuation (Figs. 2A, 2C). Twenty-five patients (19.5%) had mild BSL and 14 had dense deposits (11%). Thirteen patients (10%) had a greater nasal involvement, with more BSL or atrophy in this region. Peripheral FAF imaging revealed diffuse hypoautofluorescence in the majority of cases, with patches of definitely decreased autofluorescence (AF) in 20 patients (15.6%), and choroideremia (CHM)-like widespread patches in 2 patients (1.6%). Eleven patients (9%) had peripheral hypoautofluorescent (hypoAF) spots and 19 (15%) had optic disc pallor.

Sixty-five patients (51%) had a macular hyperautofluorescent (hyperAF) ring at age 32.6 ± 15.1 years old, with a mean area of 7.6 ± 6.2 mm^2^ OD and 8.1 ± 7.2 mm^2^ OS. Focusing on the adult cohort (> 20 years old, excluding 16 individuals), the baseline ring area was 5.9 ± 4.5 mm^2^ OD and 6.2 ± 5.5 mm^2^ OS. Macular appearance on examination and imaging was within normal limits in 68 patients (53%), with areas of atrophy in the remaining patients. There was a significant association between age and the ring area OD and OS (*P* = 0.008 and 0.03, *R*^2^ = 0.1), but not between BCVA and the ring area (*P* = 0.3 and 0.94). There was no significant difference between the area in the OD versus OS (*t*-test *P* = 0.83).

#### Sector RP and Unaffected Carriers

Sixty-three patients (87.5%) had retinal images at a mean age of 43.9 ± 17.4 years old. Fifty-nine patients (81.9%) had widefield color images and the common features were BSL and atrophy in the inferior (*n* = 26), nasal (*n* = 2), inferotemporal (*n* = 2), or inferonasal (*n* = 19) retina (Figs. 2B, 2D). Five patients (7%) had normal color images and only visible abnormalities with FAF. Peripheral FAF showed a diffuse and patchy hypoAF region in the inferior arcade, with a hyperAF macular border. Only one patient had peripheral hypoAF spots and one patient had optic disc pallor.

Twenty-one patients (29.2%) had a complete macular hyperAF ring at age 42.4 ± 14.7 years old, with an area of 6.6 ± 4.4 mm^2^ OD and 6.9 ± 4.3 mm^2^ OS. The younger patients (< 20 years old) did not have rings in FAF. Nine patients (12.5%) had macular atrophy, and the remaining patients had a normal macula. There was no significant association between age and the ring area OD and OS (*P* = 0.06), or between BCVA and the ring area (*P* = 0.3). There were no significant differences between the area in the OD versus OS (*t*-test *P* = 0.47).

#### Genotype-Phenotype Correlations

Dense BSL was more common in class 1 (8 of 45 with widefield color imaging, 18%), than in class 2 (5 of 58, 9%) and class 4 (1 of 27, 4%). Conversely, minimal or no BSL were more frequent in class 4 (43.5%), followed by class 2 (*n* = 23, 40%) and last class 1 (*n* = 10, 22%). The 2 patients with CHM-like hypoAF lesions were from class 1 and class 2.

Comparing all classes at a mean age of 38 years old, the macular hyperAF rings were significantly larger in class 2 (OD = 8.4 ± 5.3 mm^2^ and OS = 9.4 ± 6.6 mm^2^) compared to class 1 (OD and OS 5.2 ± 4.7 mm^2^, *P* = 0.03 OD and 0.02 OS). No significant differences were found between class 2 and 4, or class 1 and 4.

### Macular OCT Analysis

**Figure 3.**
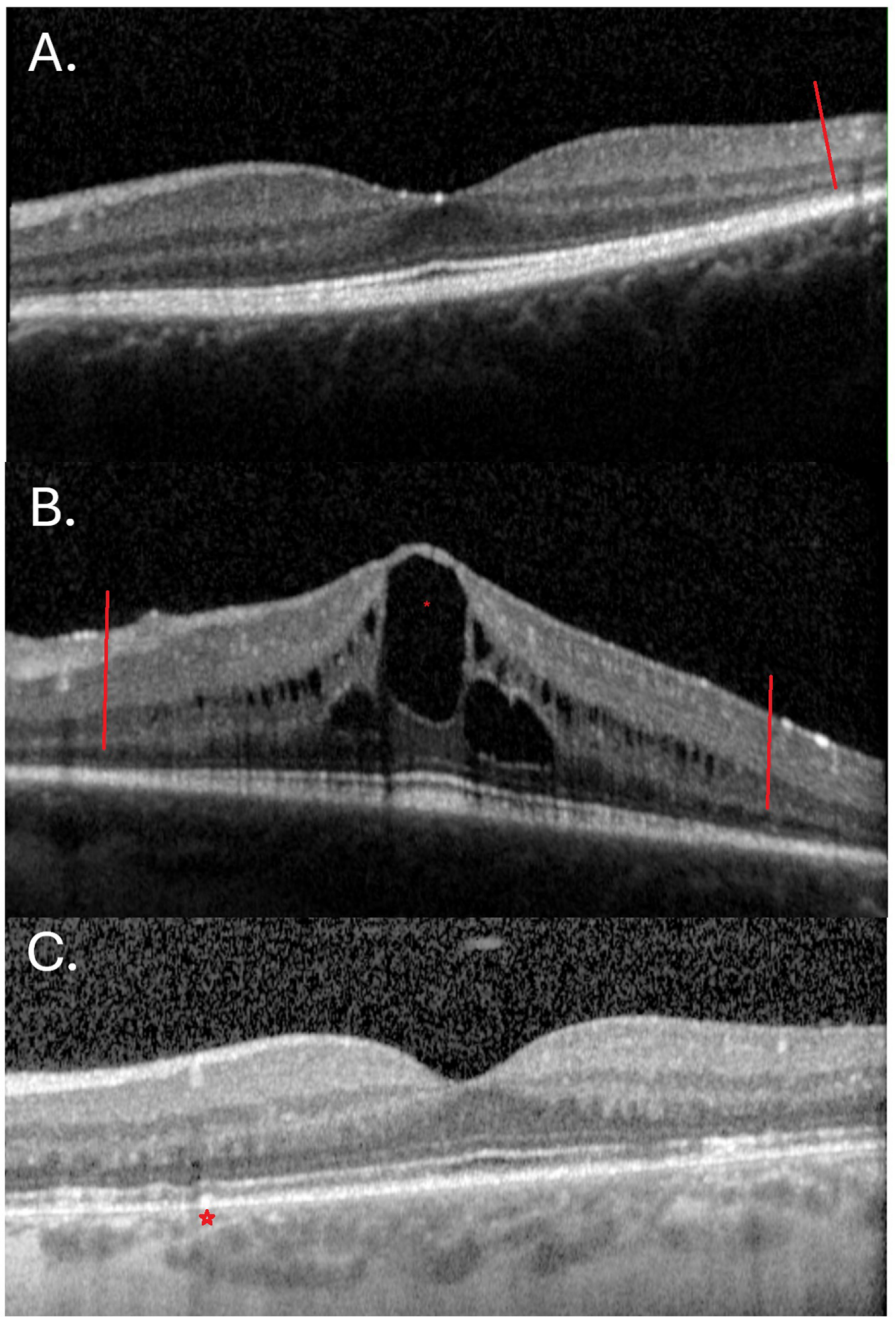
Macular OCT scans of patients with *RHO*-associated retinitis pigmentosa (RP). (**A**) The left eye of patient ID 83 with sector RP showing a largely preserved structure and a complete ellipsoid zone (EZ) line nasally at baseline (18 years old). The temporal edge is marked with a *red line*. The nasal border started to become visible 2 years later. (**B**) The left eye of patient ID 165 with generalized RP, with chronic cystoid macular edema (CMO; marked with *) altering the retinal layers and macular thickness, with a large cyst at the fovea. The EZ line also appears narrowed, with the edges marked with *red lines*. The patient had CMO since age 11 years old until the latest follow up (33 years old), despite topical and oral treatment. (**C**) The left eye of patient ID 88 with generalized RP depicting a complete EZ line, nasal drusen-like deposits (marked with a *star*) and largely normal macular scan at age 25 years old.

One hundred seventy-six patients (352 eyes) had macular OCT scans (87%); 110 with generalized RP, 64 with sector RP, and 2 unaffected carriers (see Fig. 3). Mean age at baseline OCT was 39.5 ± 17.7 years old (median = 39.5 years).

#### Generalized RP

At the baseline visit, 88 patients (69%) had an epiretinal membrane (ERM), 2 of which altered the foveal profile; 43 patients (33.5%, 72 eyes) had cystoid macular edema (CMO); and 5 eyes had macular holes. In the eyes where the foveal architecture was altered, central macular thickness (CMT) and ONLT analysis were not undertaken. One eye had subretinal fluid and was also excluded from CMT and EZ analysis.

Mean age of those with CMO was marginally younger than those without (33 ± 19 vs. 40 ± 17 years old, *P* = 0.068). Mean duration of CMO was 5.6 ± 4 years (median = 5). Eleven patients were monitored without treatment, one eye resolved, one patient did not come for follow up, and the remaining nine had ongoing CMO at their last visit. Ten patients were treated only with dorzolamide drops, one had improved CMO, two did not have follow up visits, and the remaining ones had chronic edema; three patients used brinzolamide, two had ongoing oedema, and one was not followed up; two patients had intravitreal medication (anti-VEGF and steroids), only one eye of one patient resolved; lastly, a sequential combination of drops (dorzolamide, brinzolamide, and ketorolac) and/or acetazolamide resulted in only one eye resolving and the remaining having ongoing CMO. In total, 6 eyes (8%) resolved, 58 eyes (81%) had chronic CMO, and 8 eyes (11%) did not have a follow up assessment.

Baseline CMT was 250 ± 50 µm (median = 261.5 µm), significantly decreased when compared to normative data (*P* < 0.0001; see Table 1).^17^ Mean difference between OD and OS was 0.9 ± 9.5 % (range = 0–41.1%). Mean ONLT was 87.5 ± 39.5 µm, which was similar to normative data (*P* = 0.1), with a difference between eyes of 3.1 ± 28.2% (range = 0–76.2%). The EZ line was absent in 17 eyes and full in 6 eyes (i.e. unable to assess the temporal border in 4 eyes, and the nasal border in 2 eyes; see Fig. 3). The mean EZW was 2.0 ± 1.3 mm (median = 1.9 mm) in the remaining 191 eyes, and the difference between eyes was 3.7 ± 55.3% (range = 0–366.7%). Baseline EZW of the cohort excluding 23 patients 20 years old or younger was 1.6 ± 1.2 mm (median = 1.7 mm). PROS were absent in 55 eyes and had a mean length of 12.5 ± 15.5 µm (median = 8 µm), with a difference between eyes of 2.0 ± 30.0% (range = 0–87.5%). All values (CMT, ONLT, and EZW) had a statistically significant association with age (*P* = 0.016 to < 0.0001, *R*^2^ = 0.06–0.36). There was also a significant association between BCVA and all aforementioned structural OCT parameters (*P* < 0.0001–0.0004, *R*^2^ = 0.1–0.25).

#### Sector RP and Unaffected Carriers

Thirty-eight patients (53%) had an ERM, 1 which altered the foveal profile; 35 eyes (33%, 22 patients) had CMO, and 3 eyes had macular holes. CMT and ONLT analysis were not undertaken where foveal architecture was altered. One eye had subretinal fluid and was also excluded from CMT and EZ analysis.

The mean age of those patients with CMO was older than those without (53 ± 19 vs. 39 ± 15 years old, *P* = 0.004). Mean duration of CMO was 5.4 ± 3 years (median = 5 years). Nine patients were monitored without intervention, all had ongoing cysts in the last visit (3 without follow up assessments). Five patients were treated with dorzolamide drops: one eye resolved, one patient did not have a follow up visit, and the remaining patients remained chronic. The other eight patients received a sequential combination of drops (dorzolamide, brinzolamide, and ketorolac), and/or acetazolamide, and one had an orbital floor injection of triamcinolone and methylprednisolone; only one patient had resolved CMO and the remaining had ongoing cysts. In total, only 3 eyes (9%) resolved, 24 (69%) had chronic CMO, and 8 (23%) did not have a follow up assessment.

Baseline CMT was 278.5 ± 32 µm (median = 278 µm), which was in keeping with the normative population (*P* = 0.57; see Table 1). Mean difference between OD and OS was 0.3 ± 4.2% (range = 0–13.3%). Mean ONLT was 104 ± 23 µm, which was larger than normative data (*P* < 0.0001), with a difference between eyes of 0.9 ± 12.7% (range = 0–37.4%). The EZ line was present in all eyes and full in 30 eyes (both nasal and temporal borders not captured), we were unable to assess the temporal border in 4 eyes, and the nasal border in 15 eyes. Mean EZW was 2.6 ± 1.2 mm (median = 2.6 mm) in the remaining 83 eyes, and the difference between eyes was 1 ± 24.9% (range = 0–80.2%). Baseline EZW of the cohort excluding 4 patients 20 years old or younger was unchanged (2.6 ± 1.2 mm). PROS were absent in 8 eyes and had a mean length of 17.5 ± 19 µm (median = 6.5 µm), with a difference between eyes of 4.5 ± 29.7% (range = 0–94.1%). Only EZW was significantly associated with age (*P* = 0.03, *R*^2^ = 0.1; the remaining values *P* = 0.3–0.5). BCVA was significantly associated with ONLT (*P* < 0.0001 and 0.0042, *R*^2^ = 0.3 and 0.16), EZW (*P* = 0.0009 and 0.0042, *R*^2^ = 0.25 and 0.19), and PROS length (*P* < 0.0001 and 0.0229, *R*^2^ = 0.3 and 0.08); nearly with CMT OD (*P* = 0.053, *R*^2^ = 0.07), but not for CMT OS (*P* = 0.2).

There were statistically significant differences between eyes with generalized versus sector RP regarding CMT (OD *P* = 0.002 and OS *P* = 0.009), ONLT (OD and OS *P* < 0.0001), EZW (OD *P* = 0.001 and OS *P* = 0.008), and PROS length (OD and OS *P* < 0.0001).

#### Genotype-Phenotype Correlations

Comparing all classes at a mean age of 39 years old (mean age where the classes had as many patients as possible), there were significant differences in CMT between OD class 1 (mean = 241.8 ± 52.4 µm) and class 4 (272.4 ± 36.1 µm, *P* = 0.02) variants, and near significant between class 1 and 2 (263.2 ± 45.7 µm, *P* = 0.06); whereas the other CMT comparisons were not significant (*P* = 0.19–0.48; see Table 3).

There were no significant differences in ONLT between classes (*P* = 0.38–0.77). PROS length differences were significant in OD (11.5 ± 7.9 µm) and OS (10.7 ± 8.8 µm) class 1 versus OD (15.8 ± 8.5 µm, *P* = 0.017) and OS class 2 (17.1 ± 7.1 µm, *P* = 0.004), and with OS class 4 (14.9 ± 6.8 µm, *P* = 0.03).

There were significant differences in EZW between class 1 (OD = 1559.7 ± 1073.1 µm and OS = 1521.2 ± 1089.8 µm) and class 2 (OD = 2439.4 ± 1447.2 µm, *P* = 0.002 and OS = 2521.8 ± 1380.3 µm, *P* = 0.0004) variants; and between class 1 and class 4 (OD = 2261.8 ± 1300.5 µm, *P* = 0.02 and OS = 2333.7 ± 1195.8 µm, *P* = 0.006). Remaining comparisons between classes ranged between *P* = 0.12 and 0.86. Of all the 49 patients with OCT in class 1, only 1 patient had an EZW exceeding the limits of the OCT scan (2%), compared to 18 of the 63 in class 2 (29%), and 8 of the 30 in class 4 (27%).

Regarding CMO, 18 patients in class 1 had at least one eye affected (37%), with 17 in class 2 (27%), and 8 in class 4 (27%).

### Longitudinal Analysis

One hundred sixty-one patients had follow-up assessments (80.5%), with a mean follow up time of 10.4 ± 9.2 years (median = 8 years).

#### Generalized RP

**Figure 4.**
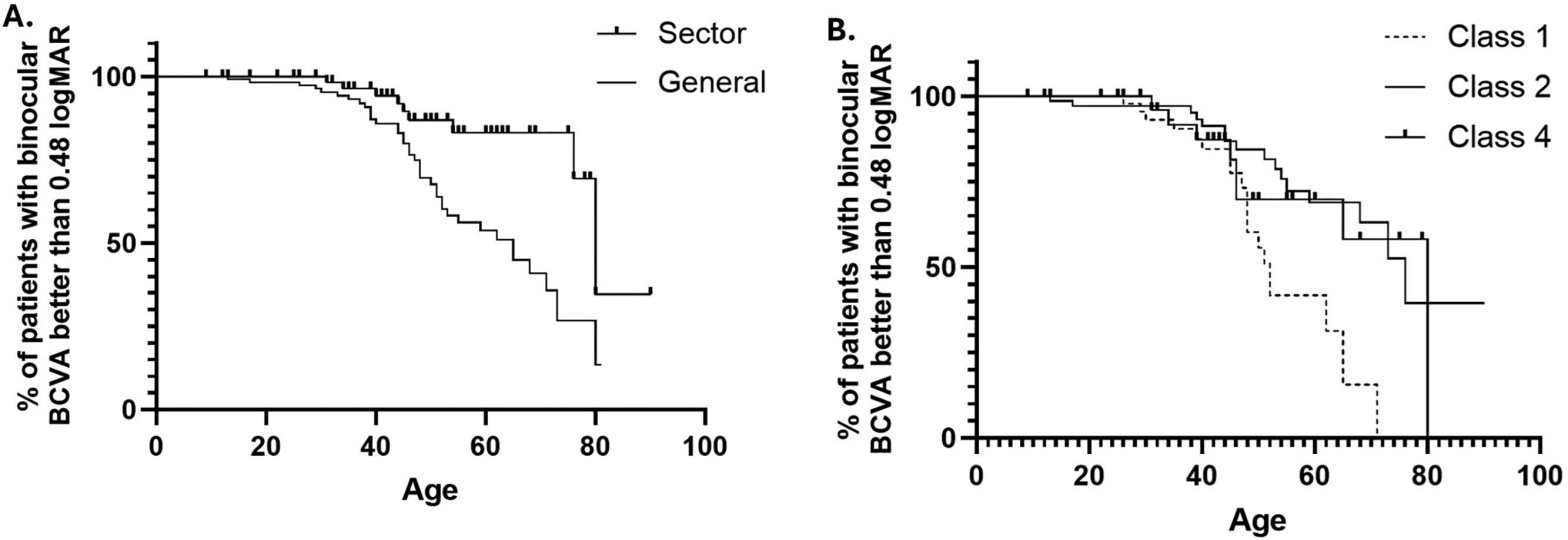
Longitudinal estimates derived from Kaplan-Meier analysis. (**A**) Generalized versus sector retinitis pigmentosa (RP), showing that by the age of 63 years, approximately 50% of the patients with generalized RP would have BCVA worse than LogMAR 0.48 (moderate visual impairment based on BCVA); whereas, for those with sector RP, this milestone would happen at around 80 years of age. There was a statistically significant difference between the types of RP (*P* = 0.009). (**B**) Analysis comparing different classes of variants. For patients with variants in class 1, there were 50% of the patients who would have BCVA worse than LogMAR 0.48 by 52 years old, for those in class 2 by 76 years old, and those in class 4 by 80 years old, with statistically significant differences between classes 1 and 2 (*P* = 0.001) and classes 1 and 4 (*P* = 0.04), but not between classes 2 and 4 (*P* = 0.26).

Mean age at the last visit was 45.2 ± 18.9 years old (median = 44 years), and the latest BCVA was 0.6 ± 0.8 LogMAR (median = 0.3). The rate of BCVA decline was 0.03 LogMAR (1.5 letters)/year and there was a significant difference between baseline and follow up BCVA OD and OS (*P* < 0.0001). Kaplan-Meier analysis showed that by the age of 63 years, approximately 50% of the patients would have BCVA worse than LogMAR 0.48 (moderate visual impairment based on BCVA; Fig. 4A). Twenty-one patients (20%) progressed to a more severe stage of visual impairment, seven of whom became blind.

During the first 5 years of follow up (*n* = 60), the rate of BCVA decline was 0.02 LogMAR (1 letter)/year for the overall cohort; 0 letters/year for the group aged ≤ 18 years old (*n* = 9); 0.01 (0.5 letters)/year for the group aged 19 to 35 years old class (*n* = 12); and 0.03 (1.5 letters)/year for the group aged 36 years and older (*n* = 39). During years 5 to 10 of follow up (*n* = 39), the rate was 0.025 LogMAR (1.25 letters)/year; patients ≤ 35 years old had a rate of 0.015 LogMAR (0.75 letters)/year (*n* = 10), and those over 35, 0.03 LogMAR (1.5 letters)/year (*n* = 29). Last, during the period of 10 to 15 years of follow up (*n* = 23), the rate was 0.02 LogMAR (1 letter)/year, with 0 for the < 35 years old (*n* = 7), and 0.03 LogMAR (1.5 letters)/year for the older subclass (*n* = 16).

Eighty-three patients had follow-up retinal imaging, at a mean age of 43 ± 18.5 (median = 43) years old. On fundus photography, denser BSL pigment was observed over time (sometimes asymmetric, in the temporal or inferior retina, *n* = 2) and more atrophic patches.

Two patients lost the hyperAF macular ring over 14 and 15 years of follow up. The final mean ring area was 6 ± 4.5 mm^2^ (median = 4.5), with a rate of ring constriction of 0.4 ± 0.8 mm^2^/year (median = 0.2). The final mean ring area for the adult cohort (> 20 years old) was 4.7 ± 3.7 mm^2^, and the rate of ring constriction, 0.3 ± 0.35 mm^2^/year. The ring area continued to be significantly associated with age (*P* < 0.0001 and 0.0002, *R*^2^ = 0.3), but not with BCVA (*P* = 0.5).

Ninety-two individuals had follow-up OCT scans at 44 ± 19 (median = 44) years old (Supplementary Fig. S6A). There were significant differences between baseline and follow up CMT (*P* < 0.0001, −1.1 µm/year), ONLT (*P* < 0.0001, −1.5 µm/year), EZW (*P* < 0.0001, −82 µm/year), and PROS length (*P* < 0.0001, −0.5 µm/year). The average rate of EZW change in the adult cohort (> 20 years old) was −79 µm/year. BCVA remained significantly associated with EZW OD (*P* = 0.0082, *R*^2^ = 0.1), but not OS or with CMT, ONLT, or PROS length (*P* = 0.4 to 0.5). Five eyes continued to have an EZ that could not be assessed due to extending beyond the OCT scan limits. Kaplan-Meier analysis showed that by the age of 58 years, approximately 50% of the patients would have an EZW under 1500 µm in both eyes, and by 65 years old, it would be under 1000 µm in both eyes.

#### Sector RP and Unaffected Carriers

Mean age at the last visit was 47.6 ± 17 years old (median = 46 years), and latest BCVA was 0.3 ± 0.5 LogMAR (median = 0.1). The rate of BCVA decline was 0.01 LogMAR (0.5 letters)/year and there was a significant difference between baseline and follow up BCVA OD (*P* = 0.009) and nearly OS (*P* = 0.06). Kaplan-Meier analysis showed that by approximately 80 years of age, 50% of the patients would have BCVA worse than LogMAR 0.48 (moderate visual impairment based on BCVA; see Fig. 4A). There was a statistically significant difference between sector and generalized RP (*P* = 0.009). Seven patients (10%) progressed to a more severe stage of visual impairment, two of whom became blind.

During the first 5 years of follow up (*n* = 30), the rate of BCVA decline was 0.01 LogMAR (0.5 letters)/year for the overall cohort; 0.03 letters/year for patients aged ≤ 40 years old (*n* = 11) and 0 letters/year for the patients 41 years and older (*n* = 19). During years 5 to 10 of follow up (*n* = 16), the rate was 0.02 LogMAR (1 letter)/year and, last, during the period of 10 to 15 years of follow up (*n* = 8), the rate was 0.02 LogMAR (1 letter)/year.

Forty-two patients had follow-up retinal imaging, at 46 ± 18 (median = 46.5) years old. Overall changes were denser BSL pigment and atrophy, mostly in the inferior retina.

The final mean ring area was 5.9 ± 4.8 mm^2^ (median = 4.6), with a rate of ring constriction of 0.6 ± 0.9 mm^2^/year (median = 0.4). The ring area continued to be significantly associated with age (*P* = 0.025, *R*^2^ = 0.3), but not with BCVA (*P* = 0.9).

Forty-nine individuals had follow-up OCT scans at 48 ± 18 (median = 47) years old (see Supplementary Fig. S6A). There were no significant differences between baseline and follow up CMT (*P* = 0.3, 0.4 µm/year), ONLT (*P* = 0.4, −0.5 µm/year), and PROS length OD (*P* = 0.12, −0.1 µm/year). There were significant differences for EZW (*P* < 0.0001, −100 µm/year) and PROS length OS (*P* = 0.002, −0.9 µm/year). The children in this group either had no longitudinal data for EZW or had full EZ at baseline or latest visit. BCVA was not significantly associated with any structural parameter (*P* = 0.1 to 0.9). Twenty-six eyes continued to have an EZW that could not be assessed due to extending beyond the OCT scan limits. Kaplan-Meier analysis showed that by the age of 79 years, approximately 50% of the patients would have an EZW under 1000 or 1500 µm in both eyes (no difference between thresholds).

#### Serial ERG Data

Serial ERG data were available in 8 patients retested between 1 and 16 years from their initial ERG (see Supplementary Fig. S5). Two of 5 patients with sector RP clinically (cases 50 and 167) showed evidence of relatively mild worsening of retinal function over periods of 6 and 4 years, respectively. ERGs showed high stability in cases 24, 36, and 93 (sector RP) over periods of 8, 7, and 5 years, respectively. Two of 3 patients with generalized RP (cases 59 and 178) showed worsening over periods of 1 year and 16 years, respectively. Case 14 (generalized RP) showed only residual dark-adapted ERGs at baseline and follow-up, with undetectable light-adapted ERGs on both occasions.

#### Genotype-Phenotype Correlations

##### Best Corrected Visual Acuity

Rate of BCVA progression was 0.025 OD and 0.035 OS LogMAR (1.25–1.75 letters)/year for class 1, and at the last visit (43.7 ± 18.3 years old, median = 45 years), 4 individuals were blind, and 3 were severely sight impaired, with a most recent BCVA of 0.6 ± 0.8 OD and of 0.7 ± 0.7 OS (median = 0.3 OU).

In class 2, the rate of BCVA decline was 0.02 (1 letter)/year, and at the last visit (50.1 ± 17.9 years old, median = 50 years), 6 individuals were blind and the latest BCVA was 0.5 ± 0.7 OD and OS (median = 0.2).

Last, in class 4, the last follow up was at 47.4 ± 16.9 years old (median = 45 years), the rate of BCVA progression was 0.03 LogMAR (1.5 letters)/year, with 3 patients blind and 3 who were severely sight impaired, and the final BCVA was 0.6 ± 0.8 OD and 0.6 ± 0.7 OS (median = 0.1 OU).

There were no significant differences among the latest BCVA in classes 1, 2, and 4.

Kaplan-Meier analysis showed that 50% of the patients in class 1 would have a BCVA worse than LogMAR 0.48 by 52 years old, those in class 2 by 76 years old, and those in class 4 by 80 years old, with statistically significant differences between 1 and 2 (*P* = 0.001) and 1 and 4 (*P* = 0.04), but not between 2 and 4 (*P* = 0.26; Fig. 4B).

##### Fundus Autofluorescence

The rate of ring constriction was 0.2 ± 0.2 mm^2^/year in patients within class 1, 0.4 ± 0.5 mm^2^/year in class 2, and 0.3 ± 0.2 mm^2^/year in class 4. There were significant differences between classes 1 and 2 (*P* = 0.009) and near significant differences between classes 1 and 4 (*P* = 0.07), with no significant differences between classes 2 and 4 (*P* = 0.28).

##### Optical Coherence Tomography

In patients from class 1, there were no statistically significant differences between the baseline and the latest OCT metrics; CMT (*P* = 0.35–0.43, −1.6 µm/year), EZW (*P* = 0.9, −64 µm/year OD and −23 µm/year OS), ONLT (*P* = 0.17–0.49, −2.7 µm/year), or PROS length (*P* = 0.6–0.7, −0.7 µm/year).

In patients with class 2 variants, there was a significant difference between the baseline and the latest PROS length OS (*P* = 0.02, −0.9 µm/year) and a near significant difference in EZW OS (*P* = 0.057, −35 µm/year). Other comparisons were not significantly different; CMT (*P* = 0.17–0.7, −2.1 µm/year OD and −4.2 µm/year OS), EZW OD (*P* = 0.5, −96 µm/year), ONLT (*P* = 0.15–0.6, −2.5 µm/year), or PROS length OD (*P* = 0.2, −0.7 µm/year).

In class 4, there was a significant difference between the baseline and the latest EZW OS (*P* = 0.03, −45.8 µm/year) and a near significant difference in EZW OD (*P* = 0.053, −110 µm/year). No other significant differences were observed; CMT (*P* = 0.2–0.3, −1.5 µm/year OD and −2.2 µm/year OS), ONLT (*P* = 0.3–0.5, −1.5 µm/year), and PROS length (*P* = 0.12, −0.7 µm/year).

There were significant differences between the latest CMT OD in classes 1 and 2 (*P* = 0.03, 229.7 ± 58.7 vs. 259.2 ± 48.9 µm), and near significant between latest EZW OD in classes 1 and 2 (*P* = 0.059, 1581.4 ± 1224.3 vs. 2201.8 ± 1667.7 µm). PROS length were also different in classes 1 and 2, with statistical significance in OS (*P* = 0.04, 9.8 ± 8.0 vs. 13.4 ± 8.2 µm), and near significance in OD (*P* = 0.058, 10.4 ± 7.8 vs. 13.6 ± 8.2 µm). Other differences were not significant (*P* = 0.1–0.99).

There were significant differences regarding the rate of EZ loss OD between classes 1 and 2 (*P* = 0.01), and classes 1 and 4 (*P* = 0.008); and between rate of PROS length decrease OS between classes 1 and 2 (*P* = 0.04) and classes 1 and 4 (*P* = 0.02). Other differences were not significant (*P* = 0.2–0.8).

Kaplan-Meier analysis of EZW showed significant differences between classes 1 and 2 (*P* < 0.0001), but not between classes 2 and 4 (*P* = 0.18), or classes 1 and 4 (*P* = 0.21). It is estimated that by the age of 53 years, approximately 50% of the patients in class 1 would have an EZW under 1000 µm in both eyes, versus 77 years old in class 2, and 65 years old in class 4.

## Discussion

*RHO*-associated retinal dystrophy has been studied worldwide and represents 3% to 6% of the families seen in the ophthalmic genetics clinic.^28^^–^^31^ It is characterized by a large phenotypic spectrum that ranges between asymptomatic or minimally affected individuals and a severe retinal dystrophy that leads to blindness. In this paper, we describe the largest cohort to date of *RHO*-associated RP, the spectrum of phenotypes and likely disease-causing variants, and the molecular mechanisms underlying the retinal degeneration. Natural history genotype-phenotype correlations and distinctive structural features are described, likely aiding clinical diagnosis. International standard visual electrophysiology is also used to detail and quantify the functional impact of specific variant types on retinal and macular function.

Similar to other reports,^32^ over 70% of patients had a positive family history of an IRD, which underscores the high penetrance of the disease. However, it is significant that 3 individuals were unaffected carriers (last seen at 17, 34, and 46 years of age), and 6% of those with generalized RP and 12% of those with sector RP were asymptomatic (with the most recent visits at ages 9 to 79 years old). In addition, symptom onset was up to the seventh decade of life for some patients (range = birth to 68 years old) and five individuals (2.5%) had confirmed de novo variants (a scenario described previously in case reports).^33^^,^^34^ The above poses challenges for family history anamnesis, reminds us to act carefully when counseling patients with RP without known family history about risks to offspring, and highlights the importance of a genetic diagnosis.

Approximately two thirds of the patients had generalized RP (64%), with the remaining third having sector RP (34.5%); a larger proportion of sector RP compared to a Japanese cohort,^35^ but similar to another European study.^36^ Imaging findings of generalized RP show great similarity to those seen in association with other RP genes; however, some clues perhaps present in a minority of patients are a greater involvement of the nasal retina, patches of definitely decreased AF, and mottled/granular pattern or peripheral hypoautofluorescent spots at the earlier stages (see Fig. 2). The CHM-like phenotype was previously described secondary to c.620T>A (p.Met207Lys),^37^ but, in the study herein, we found it in patients with other variants (p.Pro171Leu and p.Pro347Leu), possibly representing a less common presentation of *RHO*-RP. Sector RP secondary to *RHO* affects mostly the inferior or inferonasal retina, unlike *RP1* or *RPGR* which can also show changes in the temporal retina.^11^ The age of onset in this cohort was similar to *RP1* (28.8 vs. 33.1 years old), whereas male patients with *RPGR* were reported to show symptoms at an earlier age (4 to 10 years old) and other genes like *MYO7A* or *EYS* may manifest at an older age (fifth or sixth decade of life).^11^

At baseline, 32.5% of the complete cohort had CMO; a smaller percentage than the one presented by Nguyen et al. (50%), and larger than that in Audo et al. (13%).^12^^,^^36^ In this cohort, CMO was more common in *RHO*-related RP than in patients with *CERKL*-associated retinal dystrophy, in a similar proportion to that seen in patients with *ADGRV1*-related syndromic RP, and less common than in *PDE6A* and *PDE6B*.^38^^–^^41^ It is of note that, despite different treatment strategies, CMO remained chronic in 69% of the patients with sector RP and 81% of the patients with generalized RP.

Most patients presented at first visit with no or mild visual impairment; 79% with generalized RP and 94% with sector RP. As previously mentioned,^42^ autosomal dominant conditions indeed tend to be milder than X-linked or autosomal recessive. The longitudinal analysis was in keeping with this, where (for those with generalized RP) by the age of 63 years, approximately 50% of the patients would have moderate visual impairment based on BCVA (1.0 LogMAR), whereas the mean BCVA in the fourth decade of life of patients with *RPGR*-RP is already closer to 1.0 LogMAR.^43^ Although the age of moderate visual impairment appears younger than the one reported by Nguyen et al., they analyzed their cohort as a whole (including both sector and generalized RP together).^36^ The rate of BCVA loss was very low (0.01 LogMAR [0.5 letters]/year) and similar to that reported by a Belgian study,^36^ and slower than a Japanese cohort (0.05 LogMAR/year).^35^ Interestingly, the group of patients aged 36 years or older showed a minimally increased rate of letters lost, potentially indicating that vision during the first 4 decades of life decreases at a slower pace.

Multiple genotype-phenotype correlations have been previously reported, including (i) variants in the intradiscal space were the mildest and those in the cytoplasm, the most severe^44^; (ii) variants affecting the C terminus lead to a more rapidly progressive disease in terms of visual field constriction and ERG responses^45^; and (iii) that variants could be classified according to their severity, where class A had an onset early in life.^46^ The classification used in this work (based on the study by Athanasiou et al.)^6^ combines clinical and research data to predict how the variants might alter rhodopsin biochemistry and cell biology. Interestingly, it appears to have relevant genotype-phenotype implications, with significant differences among classes in terms of visual function, retinal structure, and prognosis. Patients with variants in class 1 had the most severe phenotype, with generalized RP in all cases, the earliest mean age of onset, worst baseline BCVA, and greatest rate of progression in terms of BCVA and OCT metrics. Full-field ERG phenotypes were also the most consistently severe, being undetectable in one case and showing evidence of a severe rod-cone dystrophy in others. In contrast, most of those patients in class 4 had sector RP (86%), with the oldest mean age of onset, slowest rate of progression, and mild-moderate ERG reductions, with PERG evidence of spared or relatively spared macular function in most that were tested. Although with a smaller number of patients, it is still worth highlighting that patients in class 2/3 had an even earlier age of onset, potentially pointing to a more severe phenotype when two disease mechanisms are present (see Supplementary Figs. S6, S7).

Class 2 misfolding variants are the most common and had a wide range of clinical presentation, with both generalized (54%) and sector (43%) RP (see Table 2, Supplementary Fig. S6A), as well as a highly variable age of onset and ERG phenotype. The reasons for this variability are unclear. It is possible that variants with some residual rhodopsin folding, leading to some trafficking to the outer segment, might present as sector RP, whereas those that do not are more likely to present as generalized RP. However, there was no relationship between the DMS trafficking score and sector versus generalized RP (Supplementary Fig. S8; each point is an individual separated by their phenotype and mechanistic class). In addition, some variants are associated with both forms of RP (even in the same family) suggesting that other factors might influence this, such as environmental (e.g. bright light exposure) or genetic modifiers. The rate of disease progression is unlikely to be determined by the extent of misfolding alone, as the p.Cys110Arg variant, that cannot form the correct disulphide bond and is an obligate misfolding variant, has a relatively late age of onset and slow reduction in EZW (see Supplementary Table S1, Supplementary Fig. S9). In contrast, the p.Tyr178Cys class 2 variant has a relatively early onset more comparable to class 1 variants. It has been suggested that severe disease could be a consequence of increased protein aggregation, as observed in a mouse model of this variant^47^; however, another variant, p.Gly188Arg, that aggregates in mouse retina had a later age of onset than p.Pro23His which aggregates less and both presented as sector RP.^47^ Therefore, there is not a clear association between aggregation in mouse retina and generalized versus sector RP in patients, or age of onset. This could be a consequence of differences between the mouse and human rhodopsin and/or environmental and/or genetic modifiers. Nevertheless, these data highlight there is still much to learn about the underlying pathogenesis of rhodopsin RP.

The four previously unreported variants (c.999dupC, c.937-24_943delins150, c.793T>G, and c.805_807dupGCC) are particularly relevant given small indel variants are less frequent in *RHO*,^32^ potentially aiding toward further understanding disease mechanisms. As previously reported,^48^ the only variant thought to potentially escape nonsense-mediated decay (c.190C>T, p.Gln64Ter, in ID 59) led to a mild, late onset phenotype; with unknown pathophysiology. Interestingly, patient ID 84 was heterozygous for p.Glu150Lys and had typical sector RP, with preserved EZ until the fifth decade of life, whereas patient ID 104 (homozygous for the same variant) had a generalized RP phenotype and narrower EZ island, potentially pointing at an allelic dosage effect. The most common variant in our cohort (c.1040C>T, p.Pro347Leu) was also the most common in Japanese, Italian, and Spanish cohorts,^49^^–^^51^ p.Glu181Lys (the second most frequent in this cohort) was the most prevalent in a Belgian and Dutch report,^36^ and p.Pro23His being the most common in the United States, was only found in one White patient in our cohort.^52^ Modeling the most common class 4 variant (p.Thr58Arg) in a knock-in mouse provided further evidence that bright light may act as a disease initiator/accelerator in retinal dysfunction and degeneration (see Supplementary Fig. S2). This supports the advice given to patients with RP to avoid excessive exposure to bright sunlight, including wearing good quality UVA/UVB sunglasses and a hat.

The study strengths include a large cohort of genetically proven patients with *RHO*-associated RP with longitudinal data, functional testing and multimodal imaging, and a multidisciplinary team of authors who also undertook preclinical work in vivo and in vitro to better understand this condition. The limitations include differences in follow up between patients, device operators, and versions of the devices, and unknown and/or inconsistent distance between patient's eye and the device.^53^ To mitigate measurement inaccuracies cross-sectionally in the EZW and FAF ring area due to shorter axial lengths in younger patients (compared to the assumed 24 mm by Spectralis) as well as longitudinally due to growing eyes, these were excluded from some of the analyses (where applicable). However, it is established that patients with autosomal dominant forms of RP are less severely affected than those with X-linked RP. As a result, their emmetropization mechanism may also be less severely disrupted^54^; hence, adults with *RHO*-associated RP are generally emmetropic or mildly ametropic, and the 24 mm axial length may be a reasonable estimate for our adult cohort.^55^ Additionally, the longitudinal findings of our adult cohort are unlikely to be impacted by axial length as it will remain stable over time. Results showed a narrower EZ island and FAF ring in adults, and a minimally slower rate of progression (by 3 µm and 0.1 mm, respectively), which may be explained either by a faster progression rate during younger years or by image artifact due to not scaling according to the growing axial length (or a combination of the two). Future studies including biometry data will clarify this matter.^56^ As a real-world dataset, the transverse measurements herein should be considered as estimates or a broad reference point.

Several therapeutic approaches are currently being explored for *RHO*-RP, which can be broadly divided into variant/class dependent or agnostic.^42^ The Ocugen gene therapy, OCU400, utilizes a modifier gene therapy based on delivery of the nuclear hormone receptor gene NR2E3, that might be suitable for RP secondary to multiple genes, including *RHO*, in a variant and gene independent manner. In a phase I/II clinical trial (NCT05203939), OCU400 demonstrated safety and tolerability in subjects with *RHO*-AD RP, with 80% of treated eyes (8 of 10) showing stabilization or improvement in visual function (https://ocugen.com/science-and-technology/gene-therapies/). Another gene/variant agnostic approach is the oral antioxidant N-acetyl cysteine phase III study (NCT05537220), which is enrolling patients with RP including *RHO*-RP. Aldeyra’s small molecule ADX-2191 is an intravitreal reformulation of methotrexate, used to target retinal inflammation by promoting the clearance of misfolded rhodopsin, which might be most applicable to class 2 *RHO* variants.^57^ In a phase II trial (NCT05392179), ADX-2191 demonstrated potential in improving retinal function after 3 months of treatment, with subjects showing improvement in visual acuity and other retinal functions (https://ir.aldeyra.com/news-releases/news-release-details/aldeyra-therapeutics-announces-improvement-baseline-retinal). Octant has developed an oral small molecule corrector therapy aimed at rescuing the folding and trafficking defects of misfolded rhodopsin variants associated with *RHO*-adRP. This approach is designed to target classes 2, 2/3, and 2/4 variants by stabilizing the mutant protein, reducing aggregation and facilitating its release from the ER and possible localization to the outer segments of photoreceptors. Octant is advancing its lead candidate, OCT-980, through early clinical development, with an initial phase I/IIa trial assessing safety, pharmacokinetics, and exploratory retinal endpoints in patients with *RHO*-adRP.

In conclusion, the dataset and data-driven insights herein enhance our understanding of *RHO*-RP, the disease trajectory, and the different genotypic classes. The use of this natural history data allows better counseling for patients, aiding the identification of meaningful clinical endpoints, the establishment of robust outcome measures, and participant stratification, ultimately contributing in the interpretation of therapeutic efficacy in clinical trials for patients with *RHO*-RP.

## Supplementary Material

Supplement 1

Supplement 2

Supplement 3

Supplement 4

Supplement 5

Supplement 6

Supplement 7

Supplement 8

Supplement 9

Supplement 10

Supplement 11

Supplement 12
