# Supplementary material for: RHO-Associated Retinitis Pigmentosa: Genetics, Phenotype, Natural History, Functional Assays, and Animal Model – In Preparation for Clinical Trials
Source: Invest Ophthalmol Vis Sci. 2025 Jul 30;66(9):69. doi: 10.1167/iovs.66.9.69 (PMC12315919; doi:10.1167/iovs.66.9.69)
Supplement: Supplement 1 [file iovs-66-9-69_s001.pdf]

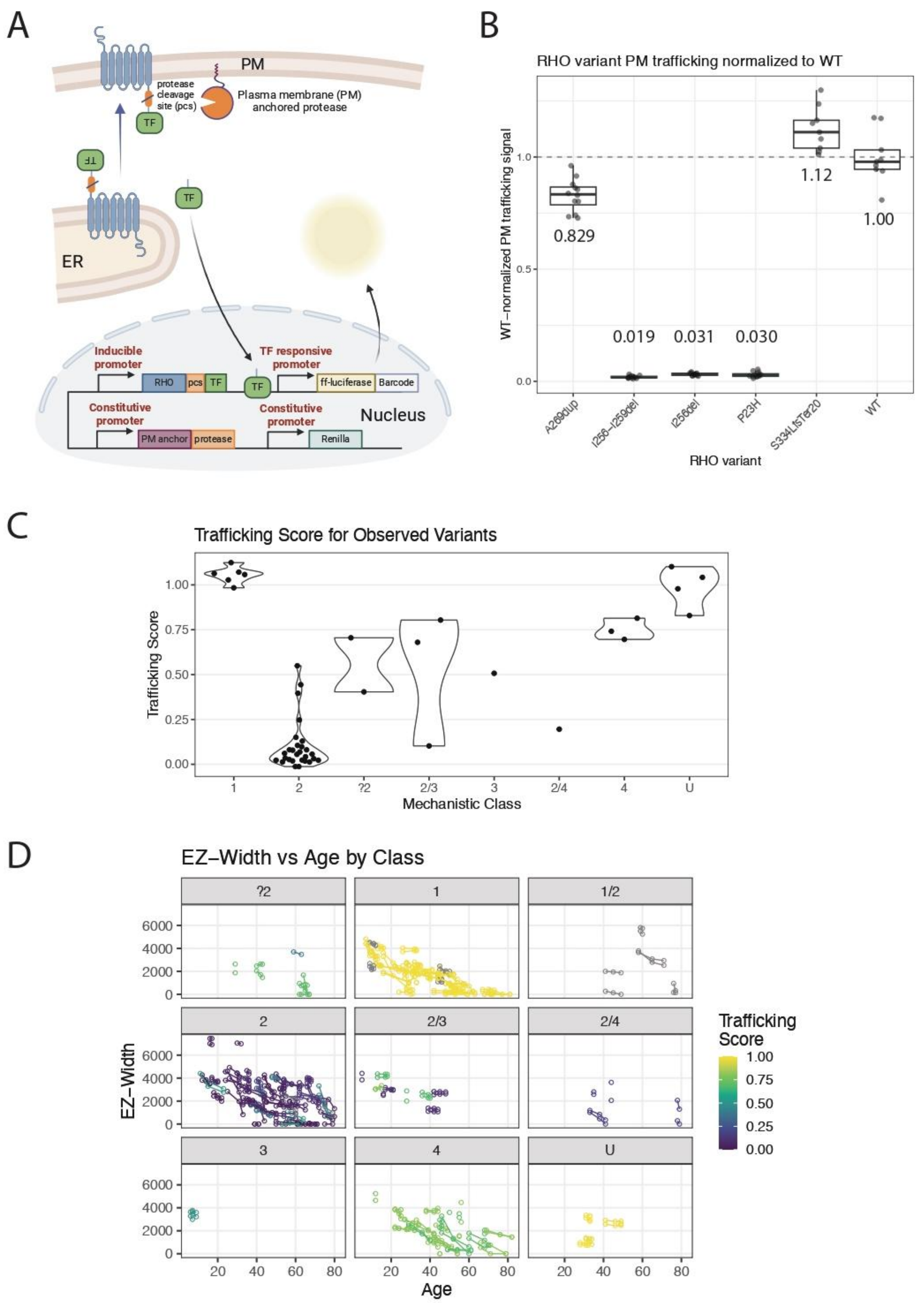

Supplementary Figure 1. (A) Schematic of the luciferase assay trafficking assay. Cells expressing RHO variants were genetically fused to a cleavable transcription factor (TF). Upon proper plasma membrane (PM) trafficking, a membrane anchored protease will cleave the TF which will activate transcription of firefly (ff) luciferase. (B) Box plot of RHO variant trafficking by luciferase assay. Raw values were normalized to the mean of WT

trafficking signal. Mean values for each variant are displayed. (C) Violin plot of each variant by its mechanistic class and trafficking score. Each point represents a unique variant. (D) Relationship between EZW and age separated by class and coloured by trafficking score (grey are variants that do not have trafficking scores). Points represent individual measurements and lines connect longitudinal measurements within an individual's eye. Class 1 variants appear to have more severe progression than Class 2 variants.
