# Supplementary material for: RHO-Associated Retinitis Pigmentosa: Genetics, Phenotype, Natural History, Functional Assays, and Animal Model – In Preparation for Clinical Trials
Source: Invest Ophthalmol Vis Sci. 2025 Jul 30;66(9):69. doi: 10.1167/iovs.66.9.69 (PMC12315919; doi:10.1167/iovs.66.9.69)
Supplement: Supplement 2 [file iovs-66-9-69_s002.pdf]

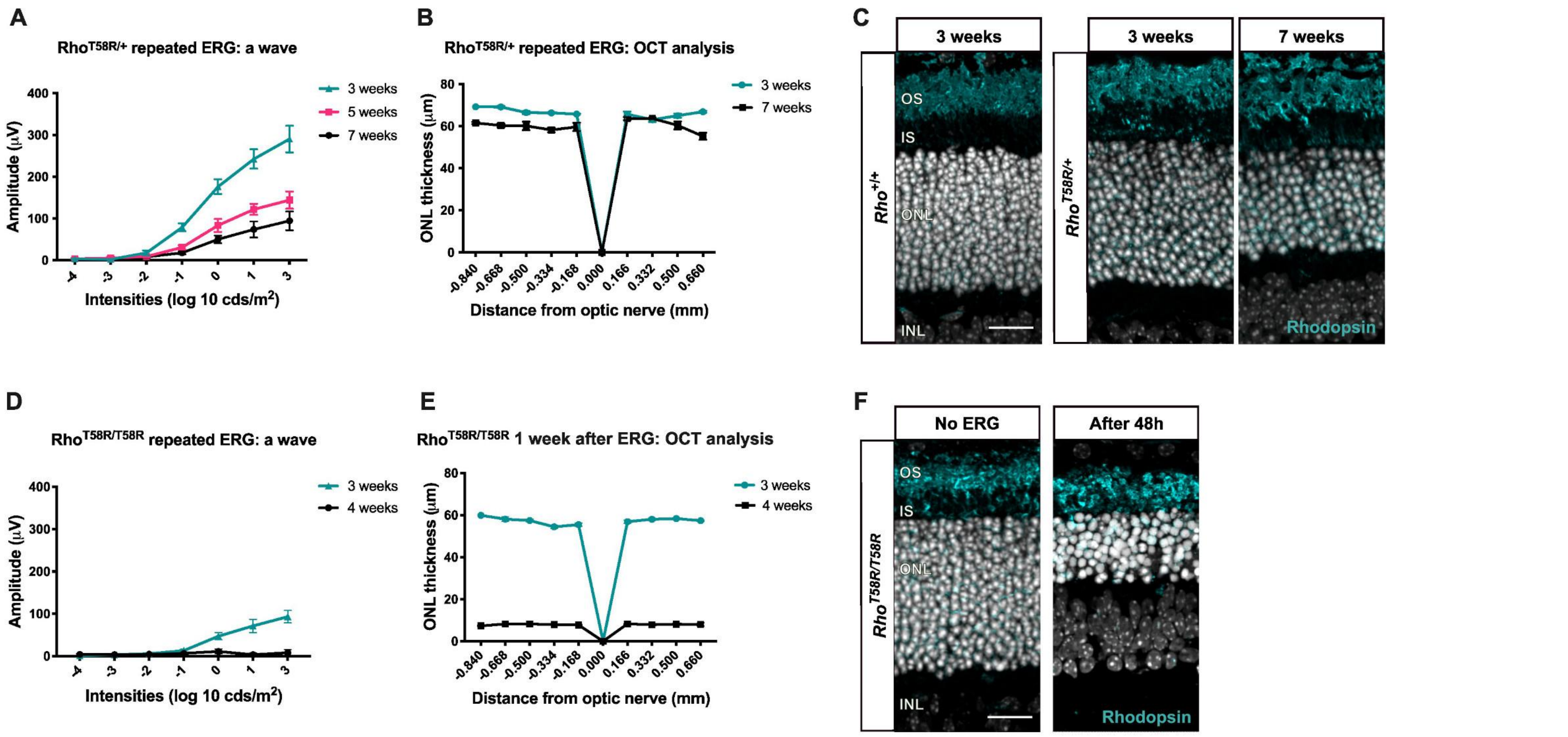

Supplementary Figure 2. Light enhanced retinal dysfunction and degeneration in Thr58Arg variant knock-in mice. Heterozygous RhoT58R/+ knock-in (KI) and homozygous RhoT58R/T58R KI mouse models were analysed by ERG, OCT and IHC. (A) The activity of the RhoT58R/+ KI mouse retina was measured every 2 weeks by scotopic ERG. The A wave reflects the photoreceptors hyperpolarization in response to different intensities of light and was plotted as positive values. Mean ± SEM, Two-way ANOVA, Tukey’s multiple

comparisons tests (**p<0.01, ***p<0.001, **** p<0.0001), N=4 mice. (B) The ONL thickness was measured by OCT in the central retina in the same mice at 3 weeks and 7 weeks. Mean ± SEM, mixed-effect analysis (****p<0.0001), N=4 mice. (C) 3 week old control (Rho+/+) and 3 and 7 weeks RhoT58R/+ retina sections were stained with anti-rhodopsin-1D4 antibody (in cyan). Scale bar = 20mm. OS = Outer Segment, IS = Inner Segment, ONL = Outer Nuclear Layer, INL = Inner Nuclear Layer. (D) The activity of the RhoT58R/T58R KI mouse retina was measured at 3 and 4 weeks on the same group of animals. Mean ± SEM, Two-way ANOVA (****p<0.0001), n=3 mice (E) The ONL thickness was also measured. Mean ± SEM, two-way ANOVA (****p<0.0001), n=3 (F) Cryosections of RhoT58R/T58R KI mouse retina before and 48hrs after ERG were stained with anti-rhodopsin-1D4 antibody. Scale bar = 20mm. OS = Outer Segment, IS = Inner Segment, ONL = Outer Nuclear Layer, INL = Inner Nuclear Layer.
