# Supplementary material for: RHO-Associated Retinitis Pigmentosa: Genetics, Phenotype, Natural History, Functional Assays, and Animal Model – In Preparation for Clinical Trials
Source: Invest Ophthalmol Vis Sci. 2025 Jul 30;66(9):69. doi: 10.1167/iovs.66.9.69 (PMC12315919; doi:10.1167/iovs.66.9.69)
Supplement: Supplement 3 [file iovs-66-9-69_s003.pdf]

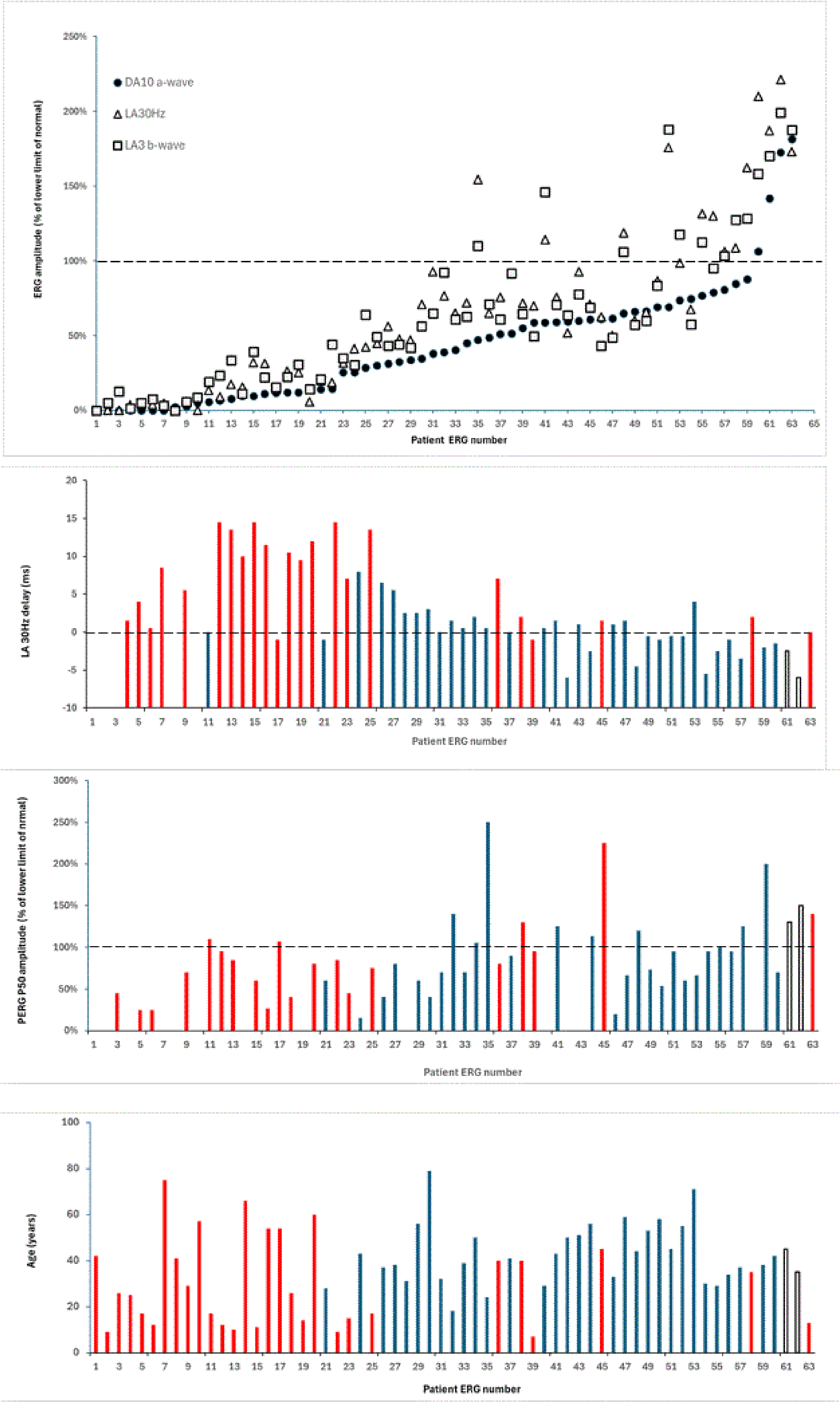

DA10 a-wave
LA30Hz
LA3 b-wave
ERG amplitude (% of lower limit of normal)
Patient ERG number
LA 30Hz delay (ms)
Patient ERG number
PERG P50 amplitude (% of lower limit of nrmal)
Patient ERG number
Age (years)
Patient ERG number

Supplementary Figure 3. Full field ERG, pattern ERG and patient ages at the time of testing, summarised in 63 individuals according the ISCEV standard methods with values arranged in ascending order of DA10 ERG a-wave amplitude for clarity. The amplitudes of the DA 10 ERG a-wave, LA 30 Hz ERG and LA 3 ERG b-wave are plotted against the percentage of the age-matched lower limit of the (“normal”) reference range (a). The LA 30 Hz peak times are plotted as a difference from the age-matched upper limit of normal timing (b). The pattern ERG P50 component amplitudes are plotted as a percentage of the lower limit of normal (c). Age of the patients at the time of testing (d). Data from patients who had a “generalised” fundus phenotype are highlighted by the red columns and data from those with a “sector” fundus phenotype are highlighted by the black columns (b, c and d).
