# Supplementary material for: RHO-Associated Retinitis Pigmentosa: Genetics, Phenotype, Natural History, Functional Assays, and Animal Model – In Preparation for Clinical Trials
Source: Invest Ophthalmol Vis Sci. 2025 Jul 30;66(9):69. doi: 10.1167/iovs.66.9.69 (PMC12315919; doi:10.1167/iovs.66.9.69)
Supplement: Supplement 4 [file iovs-66-9-69_s004.pdf]

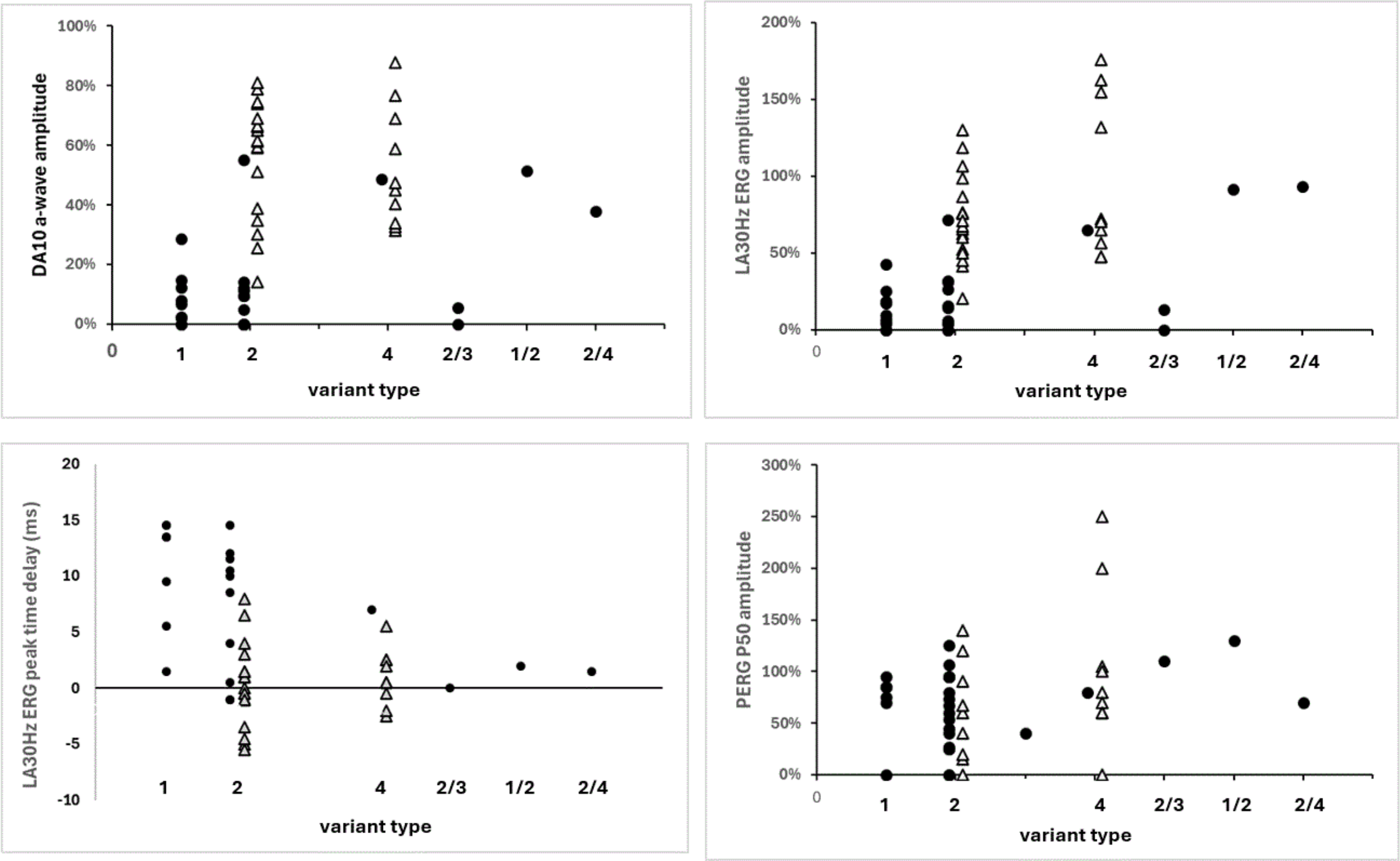

Supplementary Figure 4. Comparison of full field ERG and pattern ERG phenotypes according to variant type are shown for the DA10 ERG a-wave amplitude (a), the LA30Hz ERG amplitude (b) and peak time (c), and the pattern ERG P50 amplitude (d). Data pints associated with a "Sector RP" fundus phenotype are displaced to the right, to distinguish from those with a "generalised" fundus phenotype.
