# Supplementary material for: RHO-Associated Retinitis Pigmentosa: Genetics, Phenotype, Natural History, Functional Assays, and Animal Model – In Preparation for Clinical Trials
Source: Invest Ophthalmol Vis Sci. 2025 Jul 30;66(9):69. doi: 10.1167/iovs.66.9.69 (PMC12315919; doi:10.1167/iovs.66.9.69)
Supplement: Supplement 5 [file iovs-66-9-69_s005.pdf]

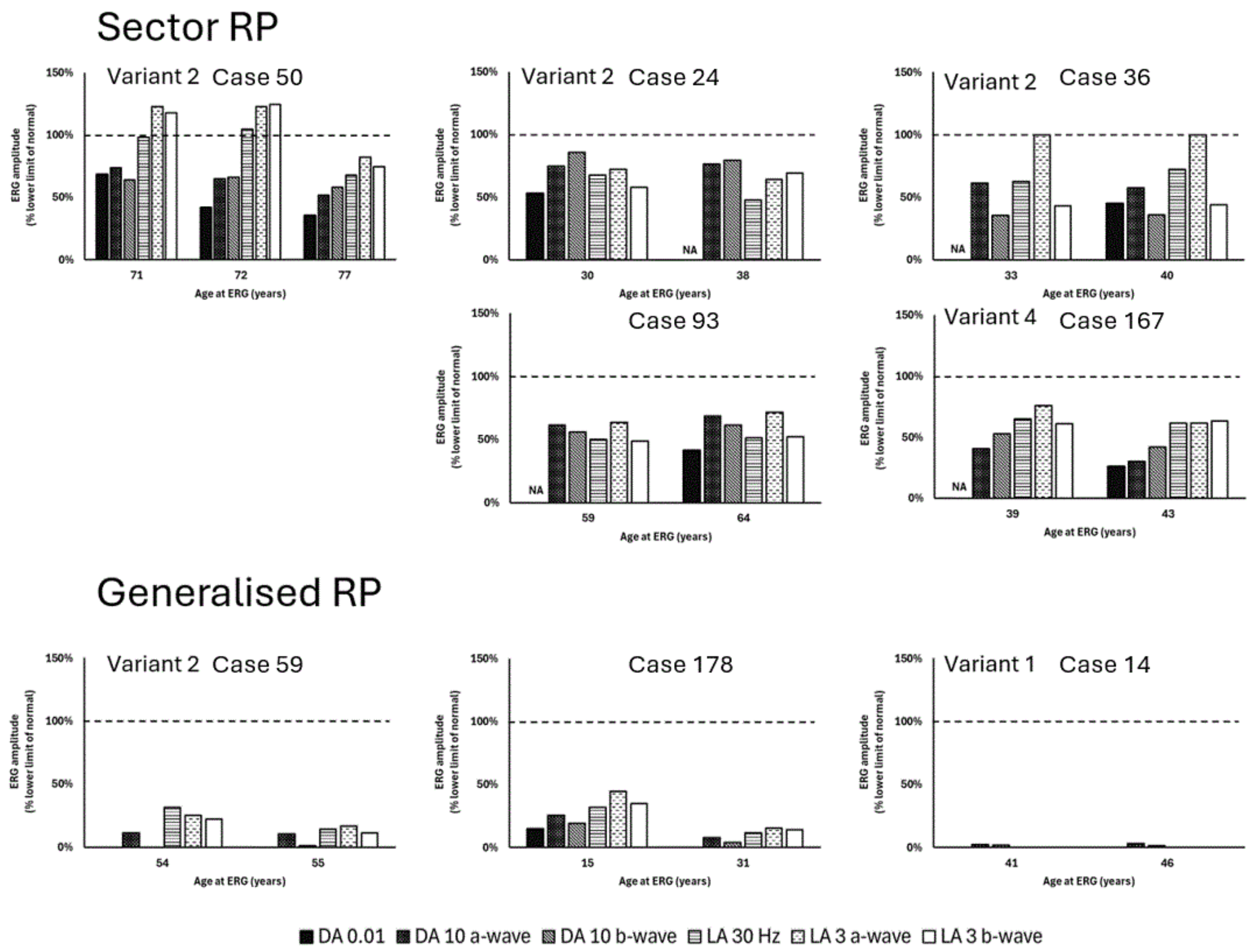

Supplementary Figure 5. Charts summarising the serial ERG data, available in 8 cases. Patients underwent baseline and repeat ERG testing over a period up to 16 years. Amplitudes are shown as a percentage of the lower age-matched reference limit. 'NA' indicates data that could not be analysed due to eye movement artefacts. In cases where the genetic variant subtype was known, these are shown in the top left corner of each graph. Broken lines indicate lower limits of the reference range.
