# Supplementary material for: RHO-Associated Retinitis Pigmentosa: Genetics, Phenotype, Natural History, Functional Assays, and Animal Model – In Preparation for Clinical Trials
Source: Invest Ophthalmol Vis Sci. 2025 Jul 30;66(9):69. doi: 10.1167/iovs.66.9.69 (PMC12315919; doi:10.1167/iovs.66.9.69)
Supplement: Supplement 6 [file iovs-66-9-69_s006.pdf]

A

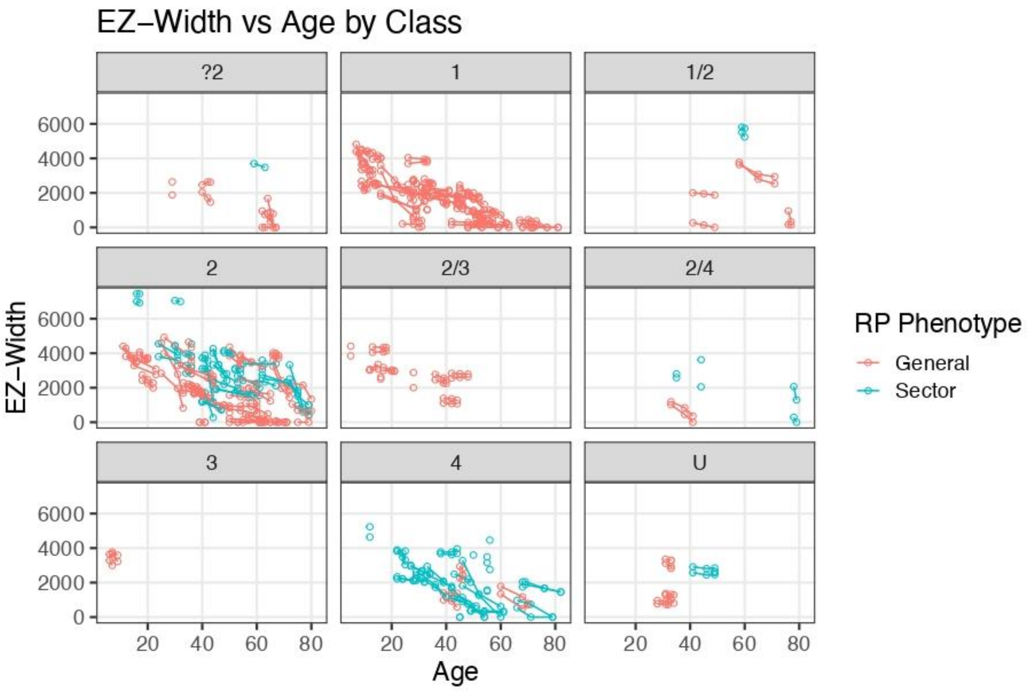

B

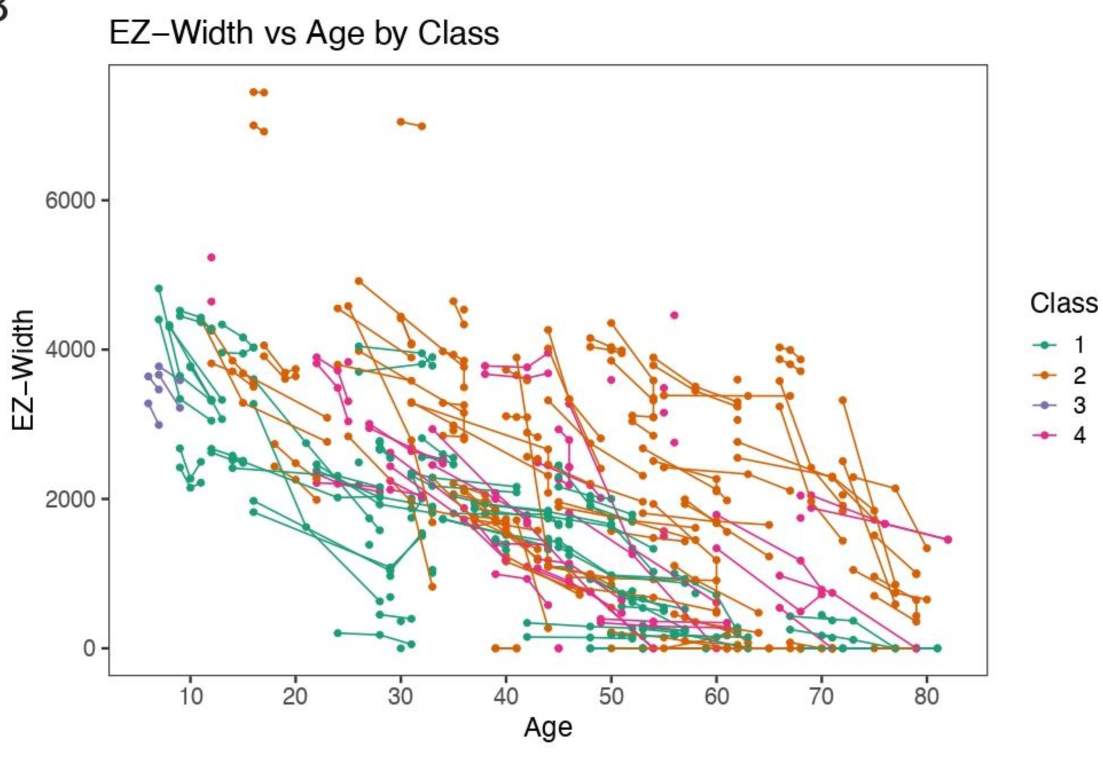

Supplementary Figure 6. (A) EZW versus age coloured by class. These data are restricted to variants that are confidently assigned to class 1-4. Points represent individual measurements and lines connect longitudinal measurements within an individual's eye. (B) EZW versus age coloured by RP phenotype separated by class. Points represent individual measurements and lines connect longitudinal

measurements within an individual's eye. Note individuals with the same RHO variant can have different RP phenotypes.
