# Supplementary material for: RHO-Associated Retinitis Pigmentosa: Genetics, Phenotype, Natural History, Functional Assays, and Animal Model – In Preparation for Clinical Trials
Source: Invest Ophthalmol Vis Sci. 2025 Jul 30;66(9):69. doi: 10.1167/iovs.66.9.69 (PMC12315919; doi:10.1167/iovs.66.9.69)
Supplement: Supplement 7 [file iovs-66-9-69_s007.pdf]

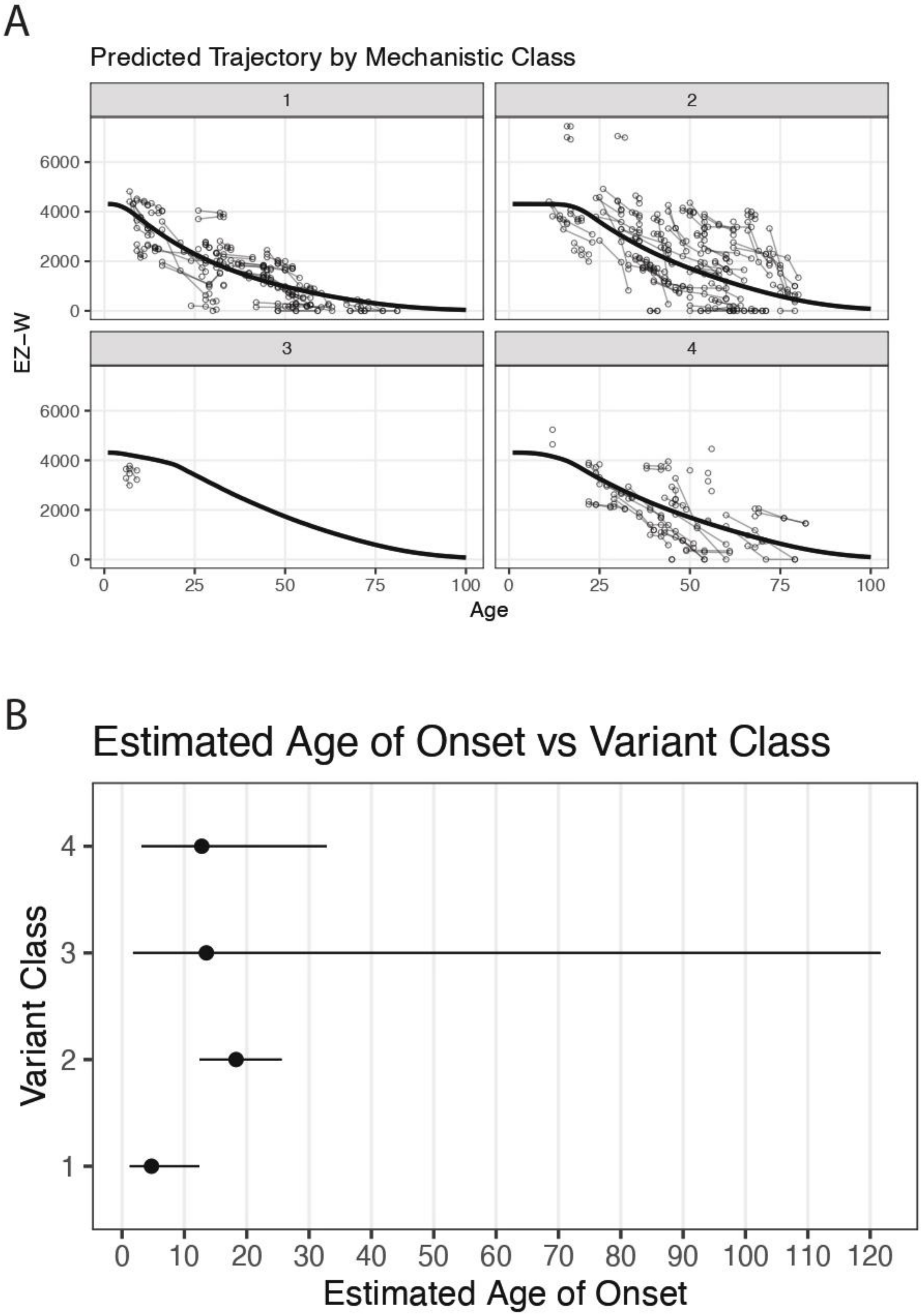

Supplementary Figure 7. (A) Predicted EZW decline for each class. We fit a non-linear Bayesian hierarchical model to variants with class 1, 2, 3, or 4 labels. This model allows EZW to stay constant for a period before exponentially declining. We take into account variant- and individual-level variation through random intercepts. We set weakly informative priors based on expectations from the literature and first principle (see

Supplementary Methods). (B) Estimated age of EZW decline onset for each class. Class 1 variants have the earliest onset of EZW decline (4.7 years old; 95% CI 1.2 - 12.4). This is significantly earlier than Class 2 variants (18.3 years old; 95% CI = 12.4 - 25.6; difference = 13.2 years; 95% Bayesian Credible Interval = 4.9 - 20.8). There was more uncertainty in estimating the Class 3 (13.5 years old; 95% CI = 1.8 - 121.7) and Class 4 (12.8 years old; 95% CI = 3.1 - 32.8), which was expected given the sparsity of Class 3 data, and the limited number of individuals with Class 4 variants under the age of 25.
