# Supplementary material for: RHO-Associated Retinitis Pigmentosa: Genetics, Phenotype, Natural History, Functional Assays, and Animal Model – In Preparation for Clinical Trials
Source: Invest Ophthalmol Vis Sci. 2025 Jul 30;66(9):69. doi: 10.1167/iovs.66.9.69 (PMC12315919; doi:10.1167/iovs.66.9.69)
Supplement: Supplement 8 [file iovs-66-9-69_s008.pdf]

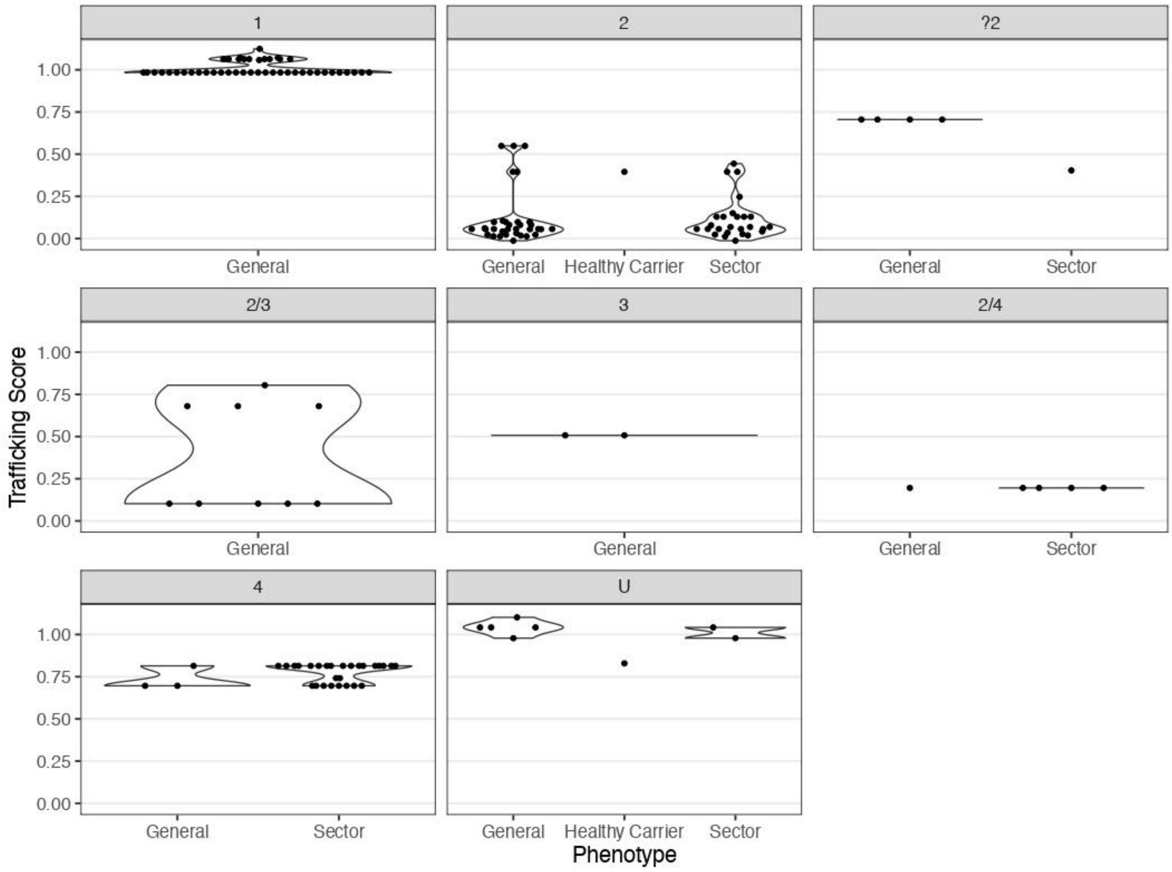

Supplementary Figure 8. Trafficking Score vs. RP phenotype by mechanistic class. Each point represents an individual. There does not appear to be any relationship between trafficking score of an individual's RHO variant and their RP phenotype across any of the classes.
