# Supplementary material for: RHO-Associated Retinitis Pigmentosa: Genetics, Phenotype, Natural History, Functional Assays, and Animal Model – In Preparation for Clinical Trials
Source: Invest Ophthalmol Vis Sci. 2025 Jul 30;66(9):69. doi: 10.1167/iovs.66.9.69 (PMC12315919; doi:10.1167/iovs.66.9.69)
Supplement: Supplement 9 [file iovs-66-9-69_s009.pdf]

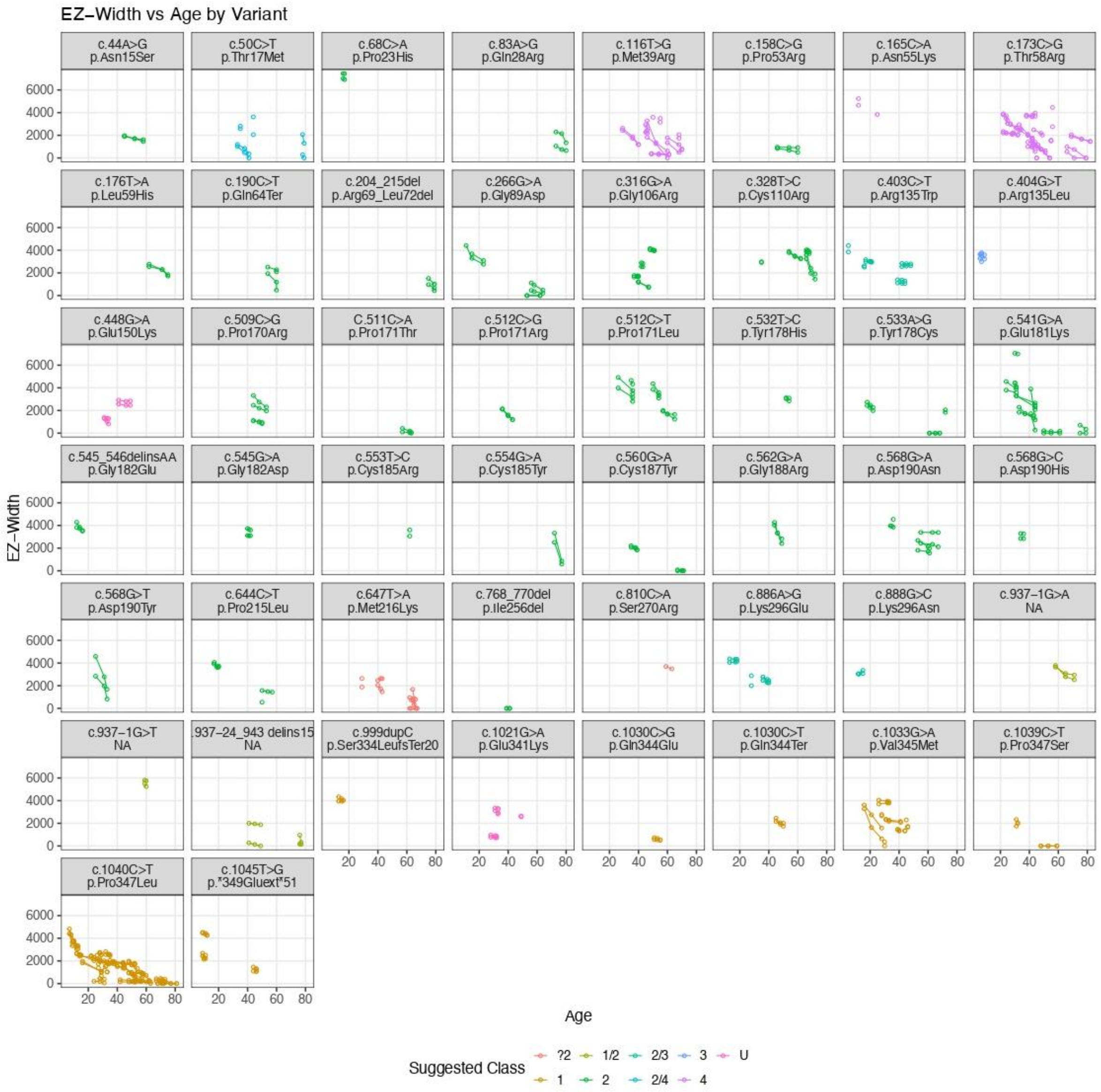

Supplementary Figure 9. EZW versus age for each variant in the dataset coloured by the proposed class. Points represent individual measurements and lines connect longitudinal measurements within an individual's eye.
