# Supplementary material for: RHO-Associated Retinitis Pigmentosa: Genetics, Phenotype, Natural History, Functional Assays, and Animal Model – In Preparation for Clinical Trials
Source: Invest Ophthalmol Vis Sci. 2025 Jul 30;66(9):69. doi: 10.1167/iovs.66.9.69 (PMC12315919; doi:10.1167/iovs.66.9.69)
Supplement: Supplement 12 [file iovs-66-9-69_s012.pdf]

Supplementary Results

Thr58Arg variant and light enhanced degeneration

The second most common variant in the cohort was c.173C>G, p.Thr58Arg (T58R) and was strongly associated with sector RP (16 out of the 19 patients with this variant, 84%), but this is relatively unstudied variant. The published reports on the effect of this variant are contradictory, with some reports suggesting ER retention, whereas others suggest trafficking is normal on heterologous expression.[1,2] Furthermore, the luciferase reporter assay showed this variant has >50% of wild-type (WT) rhodopsin traffic to the plasma membrane. Therefore, to determine the likely effects of this variant on rhodopsin *in vivo* and confirm pathogenicity we produced a knock-in mouse with the T58R amino acid substitution in the mouse *Rho* gene (Supplementary Figure 2). Repeated scotopic ERG analyses of the mice at 3, 5 and 7 weeks showed a progressive dysfunction of the rod photoreceptors (Supplementary Figure 2A). The retina of the heterozygous mice appeared similar to littermate WT mice at 3 weeks of age and rhodopsin traffic to the outer segment resembled that of WT mice (Supplementary Figure 2B), with little rhodopsin retention in the outer nuclear layer (ONL) or inner segment (IS). The animals that had longitudinal ERG assessment showed a thinning of the ONL by OCT and histology by 7 weeks of age with only a small increase in the amount of rhodopsin in the ONL and IS (Supplementary Figure 2C).

By contrast, homozygous T58R mice had a severely reduced scotopic ERG response at 3 weeks of age, suggesting that Rho-T58R is only partially functional; however, after a single ERG the scotopic ERG response was undetectable a week later. The retina of the 3-week-old homozygous mice showed good ONL preservation by OCT and histology with a most of the rhodopsin trafficking to the outer segment, with a small amount of retention in the ONL and IS. Within 48 hours of a single bright light exposure from the ERG the ONL thickness was severely reduced by immunohistochemistry (IHC) and by 7 days the ONL was almost flat (Supplementary Figure 2D-F). Collectively, these data show that T58R is not retained in the ER and can traffic to the outer segment, but that it sensitises rod photoreceptors to light induced dysfunction and degeneration. These results would support this variant being class 4 and suggest that it is a good model for sector RP, where light seems to promote degeneration in the inferior retina.

Correlation of trafficking data with mechanistic class

A previous deep mutational scan of RHO by Manian and Ludwig *et al.* measured the plasma membrane trafficking of all 6,612 possible single-residue missense variants.[3] We re-scaled the data to a 0 – 1 scale where zero represents no trafficking, and 1 represents WT-levels of trafficking. We also included variants from the luciferase-based trafficking assay (p.A269dup, p.I256del, p.I256-I259del, p.S334LfsTer20; see methods below). Similar to Manian and Ludwig *et al.*, we found the trafficking score to discriminate the different classes (Supplementary Figure 1C).

We then visualized the progression of EZW decline over time for each mechanistic class and coloured each variant by its respective trafficking score (Supplementary Figure 1D). Visual

inspection revealed noticeable differences in the progression of EZW decline between mechanistic classes. We further separated the EZW decline by all of the variants in the dataset (Supplementary Figure 1C) in order to better understand the effect of sequence variation on EZW decline.

Modelling EZW decline

To estimate both the rate of EZW decline and age of EZW decline onset, we fit a nonlinear Bayesian hierarchical model (Supplementary Methods; Supplementary Figure 6A). Briefly, this model extends previous efforts that model the EZW decay as an exponential decline by allowing for a period of "no effect" where EZW is constant followed by the exponential decline.[4] We found that Class 1 variants (4.7 years old; 95% CI = 1.2 - 12.4) had a significantly earlier age of onset for EZW decline than Class 2 variants (18.3 years old; 95% CI = 12.4 - 25.6; difference = 13.2 years; 95% Bayesian Credible Interval = 4.9 - 20.8; Supplementary Figure 6B). We also estimated the age of onset for Class 3 (13.5 years old; 95% CI = 1.8 - 121.7) and Class 4 (12.8 years old; 95% CI = 3.1 - 32.8) variants. The larger uncertainty in these estimates was expected given the sparsity of Class 3 data, and the limited number of individuals with Class 4 variants under the age of 25. Despite independently fitting the model, the estimated age of EZW decline onset were similar to the self-reported age of disease onset, indicating that structural measures of photoreceptor integrity (i.e. EZW) are a good indicator of retinal function.

	**Reported Age of Onset**	**Estimated Age of EZW Decline**
**Class 1**	13.5 ± 7.7	4.7 (1.2 - 12.4)
**Class 2**	24 ± 17	18.3 (12.4 - 25.6)
**Class 3**	Childhood	13.5 (1.8 - 121.7)
**Class 4**	29.2 ± 14.9	12.8 (3.1 - 32.8)

Supplementary Methods

Plasma membrane trafficking

Missense variant trafficking by deep mutational scan

Plasma membrane (PM) trafficking of all possible single amino acid RHO missense variants was measured by deep mutational scanning (DMS) as previously described.[3] Briefly, HEK293T cells were engineered in a pooled format to express all possible rhodopsin missense variants coupled to a variant specific reporter barcode in a similar genetic architecture as described below for the luciferase reporter. PM trafficking of all missense rhodopsin variants was measured in a pooled multiplex format by extracting RNA barcodes, preparing cDNA libraries, and performing Illumina sequencing. Variant effects were inferred by applying the negative binomial generalized linear mixed model to the sequencing count data as described in Howard *et al*.[5]

Duplication and deletion variant trafficking by luciferase reporter

Plasma membrane (PM) trafficking of rhodopsin variants p.A269dup, p.I256del, p.I256-I259del, p.S334LfsTer20, or wild-type (WT) were measured in HEK293T cells via a luciferase reporter similar to that described in [Preprint]. Briefly, HEK293T cells were stably engineered at single copy in the H11 locus to express a PM-anchored tobacco etch virus (TEV) protease and a single rhodopsin variant fused at the C-terminus to a transcription factor. The transcription factor is fused to RHO by a TEV protease cleavable linker, and upon trafficking of RHO to the PM, the transcription factor is released from RHO where it can translocate to the nucleus and activate transcription of a firefly luciferase reporter gene and a rhodopsin variant-specific RNA barcode (Supplementary Figure 1A). Cells were plated in 384 well format and measured for luciferase activity via Dual-Glo (Promega E2920) on a Tecan Infinite F Plex plate reader. A constitutively expressed renilla luciferase on the genetic cassette was measured to control for plating density (Supplementary Figure 1B).

Thr58Arg knock-in mouse production and characterisation

All procedures were conducted according to the Home Office (UK) regulations under the Animals (Scientific Procedures) Act of 1986 and with local UCL Institute of Ophthalmology, London, UK ethics committee approval. CRISPR/Cas9 gene editing was used for the creation of the rhodopsin T58R knock in mice by microinjection of Cas9 mRNA, single guide RNA (20 ng/µL) and donor into C57BL/6J embryos at the single-cell stage, as described previously.[6] The Thr58Arg (T58R) guide RNA (GTACGGTGACGTAGAGCGTGAGG) was used in combination with a donor template (TCCATGCTGGCAGCGTACATGTTCCTGCTCATCGTGCTGGGCTTCCCCATCAACTTtCTCAgGCTCTACGTCACCGTACAGCACAAGAAGCTGCGCACACCCCTCAACTACATCCTGCTCAAC) with the two lower case letter indicating the T58R change (C>G) and a silent PAM site change (CCT >TCT). Sanger sequencing was used to identify founder mice (F0), and the founders were crossed with C57BL/6J mice for at least four generations to reduce any CRISPR off-target effects.

Electroretinogram (ERG): Mice were dark-adapted overnight and anaesthetised before starting the procedure. Pupils were dilated with topical application of 1% tropicamide (Bausch & Lomb, UK). Electroretinography (ERG) was conducted using a Celeris machine (Diagnosys LLC, UK). Flash stimuli (0.1 ms to 30 ms duration, repetition rate 0.2 Hz) were delivered by the stimulator to assess the scotopic activity of the retina. Responses of a- and b-waves were recorded, and the data were exported and analysed by Excel (Microsoft) and Prism/GraphPad (Dotmatics).

Optical coherence tomography (OCT): Retinae of anaesthetised animals were imaged using the Bioptigen Envis R2300 Spectral-domain ophthalmic imaging system (SDOIS), as previously described (Athanasiou et al., 2017 PMID: 28065882). Manual segmentation of the retinal layers was performed using the Bioptigen InVivoVue Diver 2.0 software to measure the outer nuclear layer (ONL) thickness. Exported results were analysed by Excel (Microsoft) and Prism/GraphPad (Dotmatics).

Immunohistochemistry (IHC): Mouse eyes were enucleated and immersed in a 4% PFA solution overnight at 4°C. Post-fixation eyes were cryoprotected by incubation in 30% sucrose in PBS. The eyes were embedded in OCT Embedding matrix (Cell path) and cryo-sectioned at 10 μm thick sections. Sections were stained with mouse anti-rhodopsin (1D4, 1:1000) primary antibody in blocking buffer (10% serum, 3% BSA in 0.1% triton-X PBS), and visualised with secondary antibody Alexa FluorTM 488 (Goat anti-Mouse, Invitrogen, 1:1000. 4',6-diamidino-2-phenylindole (DAPI) staining was used to visualise the nuclei. Images were acquired using a LSM700 laser-scanning confocal microscope (Carl Zeiss) and processed using Image J (National Institute of Health, Bethesda, MD, USA) and Inkscape (Inkscape Project).

EZW Decay Modeling

We modelled EZW decline with a custom nonlinear Bayesian hierarchical model. Departing from the standard exponential decay model

$$\text{EZW}(\text{age}) = \text{top} \times \exp(-\text{tau} \times \text{age})$$

we allowed EZW to remain constant until a decay “switch-on.” Our formulation is

$$\text{EZW}(\text{age}) = \text{top} - \exp(\text{tau}) \times (\text{age} - \exp(\text{nec})) \times \text{I}\{\text{age} > \exp(\text{nec})\}$$

where top is the baseline EZW at age zero, exp(tau) represents the decay rate, and exp(nec) is the age at which decay commences. The indicator function I{age > exp(nec)} ensures that decline begins only after the onset age.

Both tau and nec were modelled with fixed effects of mechanistic class (coded as “class” below) and random intercepts for variant-level (aa_change) and individual-level (id) variation. This enabled us to compare the posterior estimates of Class 2 variants to the other classes for both the rate of decay (tau) and the age of onset (nec). Given the previously observed high

degree of correlation between EZW rates of decline between eyes within individuals, we chose to ignore differences in eyes. Finally, we assumed top was invariant across classes and estimated as a single intercept. In shorthand, we specified the predictors as:

tau ~ class + (1|aa_change) + (1|id)

nec ~ class + (1|aa_change) + (1|id)

top ~ 1

Because EZW values are continuous and bounded at zero, we assumed they are distributed as a hurdle–gamma distribution. Positive EZW values follow a gamma model with a log link, whereas the zero outcome is governed by a hurdle component modelled with a logit link and a linear predictor (Intercept + age). Our priors on the hurdle parameters force the probability of a zero EZW at age 0 to be negligible and to rise with age (approximately 50% probability of a zero EZW at age 80 and 88% at age 100).

We chose the following priors for the non-linear parameters of the fit based on first principles and expectations from the literature

top ~ Normal(8.5, 0.2)

Intercept for tau ~ Normal(–3.5, 0.2) and other tau coefficients ~ Normal(0, 1)

Intercept for nec ~ Normal(log(18), 0.2) and other nec coefficients ~ Normal(0, 1)

These priors give 95% density to the maximum EZW between roughly 3300–7300; the rate of decay intercept between 2 and 4.5% per year; and the age of onset intercept between 12 and 26.8 years old. The hurdle component was given priors of Normal(–8, 1) for its intercept and Normal(0.1, 0.2) for its age slope, and the gamma shape parameter received a Normal(3, 1) prior (restricted to positive values). Finally, we used the default Student_t(3, 0, 2.5) priors for the random effect terms.

We fit the model in R using brms and the cmdstanr backend with 4 chains with 2000 warm-up samples and 2000 post-warm-up samples (https://mc-stan.org/cmdstanr/authors.html#citation).[7]

References

1. Roushar FJ, McKee AG, Kuntz CP, et al. Molecular basis for variations in the sensitivity of pathogenic rhodopsin variants to 9-cis-retinal. *J Biol Chem*. 2022;298(8):102266. doi:10.1016/j.jbc.2022.102266

2. Behnen P, Felline A, Comitato A, et al. A Small Chaperone Improves Folding and Routing of Rhodopsin Mutants Linked to Inherited Blindness. *iScience*. 2018;4:1-19. doi:10.1016/j.isci.2018.05.001

3. Manian K V, Ludwig CH, Zhao Y, et al. A comprehensive map of missense trafficking variants in rhodopsin and their response to pharmacologic correction. *bioRxiv*. Published online January 1, 2025:2025.02.27.640335. doi:10.1101/2025.02.27.640335

4. Heyang M, Warren JL, Ocieczek P, et al. Long-term natural history of ellipsoid zone width in USH2A-retinopathy. *Br J Ophthalmol*. 2025;109(3):383 LP - 390. doi:10.1136/bjo-2024-325323

5. Howard CJ, Abell NS, Osuna BA, et al. High resolution deep mutational scanning of the melanocortin-4 receptor enables target characterization for drug discovery. Published online 2025. doi:10.7554/elife.104725.2

6. Arno G, Agrawal SA, Eblimit A, et al. Mutations in REEP6 Cause Autosomal-Recessive Retinitis Pigmentosa. *Am J Hum Genet*. 2016;99(6):1305-1315. doi:10.1016/j.ajhg.2016.10.008

7. Bürkner PC. brms: An R Package for Bayesian Multilevel Models Using Stan. *J Stat Softw*. 2017;80(1 SE-Articles):1-28. doi:10.18637/jss.v080.i01
